# Human Male Meiotic Sex Chromosome Inactivation

**DOI:** 10.1371/journal.pone.0031485

**Published:** 2012-02-15

**Authors:** Marieke de Vries, Sanne Vosters, Gerard Merkx, Kathleen D'Hauwers, Derick G. Wansink, Liliana Ramos, Peter de Boer

**Affiliations:** 1 Department of Obstetrics and Gynaecology, Radboud University Nijmegen Medical Centre, Nijmegen, The Netherlands; 2 Department of Human Genetics, Radboud University Nijmegen Medical Centre, Nijmegen, The Netherlands; 3 Department of Urology, Radboud University Nijmegen Medical Centre, Nijmegen, The Netherlands; 4 Department of Cell Biology, Nijmegen Centre for Molecular Life Sciences, Radboud University Nijmegen Medical Centre, Nijmegen, The Netherlands; Wellcome Trust Centre for Stem Cell Research, United Kingdom

## Abstract

In mammalian male gametogenesis the sex chromosomes are distinctive in both gene activity and epigenetic strategy. At first meiotic prophase the heteromorphic X and Y chromosomes are placed in a separate chromatin domain called the XY body. In this process, X,Y chromatin becomes highly phosphorylated at S139 of H2AX leading to the repression of gonosomal genes, a process known as meiotic sex chromosome inactivation (MSCI), which has been studied best in mice. Post-meiotically this repression is largely maintained. Disturbance of MSCI in mice leads to harmful X,Y gene expression, eventuating in spermatocyte death and sperm heterogeneity. Sperm heterogeneity is a characteristic of the human male. For this reason we were interested in the efficiency of MSCI in human primary spermatocytes. We investigated MSCI in pachytene spermatocytes of seven probands: four infertile men and three fertile controls, using direct and indirect *in situ* methods. A considerable degree of variation in the degree of MSCI was detected, both between and within probands. Moreover, in post-meiotic stages this variation was observed as well, indicating survival of spermatocytes with incompletely inactivated sex chromosomes. Furthermore, we investigated the presence of H3K9me3 posttranslational modifications on the X and Y chromatin. Contrary to constitutive centromeric heterochromatin, this heterochromatin marker did not specifically accumulate on the XY body, with the exception of the heterochromatic part of the Y chromosome. This may reflect the lower degree of MSCI in man compared to mouse. These results point at relaxation of MSCI, which can be explained by genetic changes in sex chromosome composition during evolution and candidates as a mechanism behind human sperm heterogeneity.

## Introduction

During mammalian male meiosis, the heteromorphic sex chromosomes (X and Y) condense in a separate chromatin domain known as the XY or sex body (reviewed in [1]). XY body formation starts at the zygotene to pachytene transition of prophase I [1] and persisting DNA double strand breaks (DSBs), made by SPO11 at the onset of meiosis, contribute to its formation [2]. Autosomal chromosomes are able to repair these breaks by searching for their homolog, subsequently forming a so-called bivalent in which paternally and maternally derived chromatids are closely aligned, intermediated by the synaptonemal complex (SC). However, the X and Y chromosomes are largely heterologous and show homologous synapsis only at the small pseudoautosomal region (PAR). Non-synapsis of chromosomal axial elements is detected by BRCA1 which localises ATR, a member of the PI3-like kinase family, to the X,Y chromosomes, followed by phosphorylation of H2AX at serine 139 [3]. Hence, from its formation on, the XY body is positive for γH2AX [4].

Phosphorylation of H2AX initiates repression of genes on the sex chromosomes [5], a process called meiotic sex chromosome inactivation (MSCI). Transcriptional repression of the XY body was first identified by ^3^H-uridine incorporation [6], [7], and further explored by (Cot-1) RNA FISH [8] and microarray analysis [5], [9], [10]. MSCI possibly occurs to prevent activation of the pachytene checkpoint at stage IV/V of the seminiferous epithelium in the mouse [11], [12]. MSCI is supposed to be a general phenomenon among mammals (mouse, hamster [13]) including the human [14], [15], and insects [13] and is observed in bird species as well [16]. A similar silencing process can take place at autosomal chromatin, referred to as meiotic silencing of unsynapsed chromatin (MSUC) and functions in the elimination of aberrant meiocytes bearing asynaptic autosomes [17]. MSCI is thought to be an evolutionary derivation of the more general process of MSUC [8].

Probably as a result of H2AX phosphorylation, many DNA repair proteins are attracted [18], [19] and major changes take place at XY body chromatin, including histone posttranslational modifications (PTMs) and incorporation of histone variants [20], [21]. In a previous study, we have shown for mice that specifically at X,Y chromatin and starting in early pachytene, nucleosomes containing H3.1/3.2 are replaced by nucleosomes containing H3.3 [22]. Nucleosome eviction is complete at the end of mid pachytene (stage V) [22]. Existing Histone 3 and 4 N-tail PTMs disappeared in this process and were only selectively placed back [22], allegedly to create a sex chromosome-specific chromatin composition that most likely has a function in MSCI and, by transmission over the meiotic divisions to round spermatids [22], in post-meiotic sex chromosome repression (PSCR [20]) [9]. Microarray analysis, followed by RT-PCR, demonstrated that 87% of X genes repressed during MSCI remain repressed in PMSC (post meiotic sex chromatin) [9]. The fact that in the mouse MSCI and PSCR share several features, like the same repressive histone markers and the presence of H3.3 containing nucleosomes, indicates that PSCR is a downstream consequence of MSCI.

In the human XY body, features such as a condensed chromatin domain [23] and staining for γH2AX, BRCA1 [24] and ATR [25], were shown to be present as well. Also an indication for H3.1/3.2 nucleosome eviction was obtained [22]. Cytochemical tests (acridin-orange, methylgreen pyronin) [23] and autoradiography with ^3^H-uridine incorporation [14], [15] show the absence of RNA in the human XY body thereby indicating MSCI to be present in the human. However, in a recent whole testis cDNA array analysis, MSCI could be demonstrated for the chimpanzee, but not for the human [26].

In the mouse there are extensive data documenting the association of unsynapsed autosomal segments with the XY body at first meiotic prophase. In these chromosome mutants, prophase progression, the meiotic divisions, and spermiogenesis are affected, resulting in a reduction of the number of round spermatids and a deviant heterogeneous sperm population as to morphology and motility [10], [27]–[29]. A number of observations in these mutants suggest MSCI to be compromised by the MSUC that now takes place in association with MSCI (RNA autoradiography [30] gene specific RNA FISH, [29] RNA chip analysis [10] and incomplete eviction of H3.1/H3.2 containing nucleosomes [22]). These data indicate that in mouse proper MSCI is essential for the progression of pachytene, first meiotic prophase/metaphase and influences spermiogenesis. In mice with complete avoidance of MSCI, all spermatocytes are removed by the pachytene checkpoint at the mid to late pachytene transition [12]. In human carriers of translocations a similar association of asynaptic autosomal chromosome segments to the XY body at pachytene is observed. In all cases, these men were infertile [24] (reviewed in [31]).

Additional support for the effect of disrupted XY expression on spermiogenesis in mouse comes from two sources: a) In the *Hr6b* knockout model which is characterised by poor spermatid elongation, hence male sterility, there is a general increase in post-meiotic transcription for X-linked genes [32], [33], b) In a series of Y long arm deletions, from two third to complete, post-meiotic sex chromatin showed more deviant histone and chromatin parameters when the deletion was bigger [34], in line with upregulation of X,Y genes in round spermatids [35]. Spermatid nucleus chromatin condensation is affected and so is, for the more severe deletions, the frequency of karyotype abnormalities in embryos generated by intra cytoplasmic sperm injection (ICSI) [36].

Here we have studied cytological parameters of MSCI in man over a number of probands. The main reason for this study is that human sperm samples are known to be variable between and within men [37], [38] a variability that is increasing when sperm numbers are decreasing [39], [40]. Hence, the spermiogram (number, morphology, and motility of spermatozoa) of many human probands resembles that of mouse chromosome mutants in which impaired MSCI is indicated, a variability never seen in chromosomally normal mice. A second reason is that the spermatogenic phenotypes of men with AZFc deletions suggest the Y chromosome to be involved in spermatocyte development: If MSCI is systematically operative in our species, one would not expect the deletion to be of consequence for meiocyte survival.

As a marker for MSCI we studied H3.1/3.2 nucleosome eviction. Variation was observed in the degree of nucleosome eviction in late pachytene spermatocytes. Complementary to nucleosome eviction and accompanying histone N-tail post translational modifications, we used Cot-1 RNA FISH and incorporation of the uridine analog 5-ethynyluridine into newly transcribed RNA as direct transcription markers. Our results point at both intra- and inter-proband heterogeneity in MSCI and at the absence of meiotic selection steps in the generation of round spermatids. In the discussion, we relate our observations to the roles of the sex chromosomes in spermatogenesis and to the influence of mating systems on sperm heterogeneity.

## Results

### Variation in H3.1/3.2 nucleosome eviction in XY bodies of human pachytene spermatocytes

**Figure 1:**
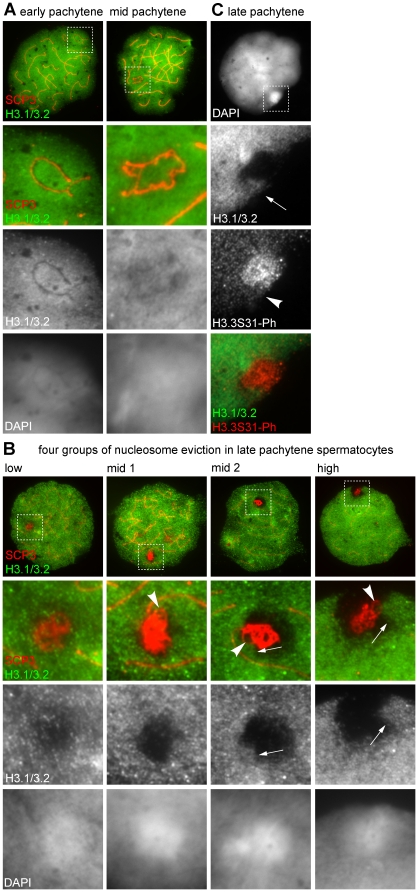
H3.1/3.2 nucleosome eviction in human pachytene spermatocytes. **A** H3.1/3.2 nucleosome eviction was never observed in early pachytene spermatocytes (Type I,II [25], [41]). First signs of H3.1/3.2 loss were detected in mid pachytene spermatocytes. **B** Illustration of four degrees of nucleosome eviction distinguished in late pachytene XY bodies. Arrows indicate the bulge of H3.1/3.2 invading the XY body. Arrowheads indicate the axial element loop reaching towards the H3.1/3.2 bulge. **C** Simultaneous loss of H3.1/3.2-containing nucleosomes and formation of nucleosomes containing H3.3S31ph at meiotic sex chromatin. Arrow indicates the H3.1/3.2 bulge, arrowhead points at the absence of enrichment for H3.3S31ph at the H3.1/3.2 bulge signal.

We examined primary spermatogenic cell samples of 3 patients and 3 controls which were obtained from testicular biopsies (the controls were of proven fertility, see Materials and Methods; Table S1). To study nucleosome eviction in human XY bodies, we stained pachytene spermatocytes for histone isoforms H3.1/3.2 and SYCP3 (as a marker for the SC and X,Y axial elements). Staining patterns were recorded by immunofluorescence (IF) microscopy. A first sign of H3.1/H3.2 loss was observed at mid pachytene when the XY axial elements locally start to unravel. (type III, [25], [41] (Figure 1A). In late pachytene XY bodies (types IV and V [25], [41]), when the XY axial elements appeared as a tangled knot of fibrils and DAPI was most intense, a highly variable H3.1/3.2 signal remained (Figure 1B, Table 1). We categorized the signals in four classes based on increasing intensity and coverage (see Materials and Methods). A minority of nuclei with a complete loss of signal for H3.1/3.2 containing nucleosomes were found (Figure 1B, Table 1), indicating a high degree of H3.1/3.2 remodelling. Remarkably, in these nuclei the border of the XY body was not as sharply defined as in our mouse samples [22]. Also between individuals in the patient group and in the control group, variation in H3.1/H3.2 staining was observed. When group data were pooled, no difference was observed between the degree of nucleosome eviction in controls versus patients (Table 1). The nuclear localization of the XY body was determined for all pachytene spermatocytes analyzed in Table 1 to investigate if there is a relation between nuclear localization and H3.1/3.2 nucleosome eviction. Figure S1 shows that XY bodies of nuclei belonging to classes ‘low’ and ‘mid 1’ nucleosome eviction are less often localized at the border of the nucleus compared to XY bodies of nuclei with a higher degree of nucleosome eviction (classes ‘mid 2’ and ‘high’). However, in every nucleosome eviction class, all possible XY body locations are represented. This indicates that our results are not a reflection of a technical artefact, such as overlaying autosomal chromatin that by DAPI and SYCP3 staining was judged to be rare: all chromatin domains are in one focal plane.

**Table 1:** Degree of sex chromatin H3.1/3.2 nucleosome eviction in patients and control men.

	Distribution of late pachytene spermatocytes among low to high nucleosome eviction classes (%)				
	Low	Mid 1	Mid 2	High	n
P1	39	27	26	8	51
P2	24	31	30	15	46
P3	48	15	15	22	60
**Total** 1	**37**	**24**	**24**	**15**	**157**
C1	43	24	28	5	61
C2	54	29	17	0	59
C3	27	22	22	28	49
**Total** 2	**42**	**25**	**22**	**11**	**169**

Patients (P), Controls (C).

1 Heterogeneity between patients χ^2^ df. 6: 13.6, p = 0,035.

2 Heterogeneity between controls χ^2^ df. 6: 31.2, p<0.001.

Pooled patients and controls: χ^2^ df. 3: 2.2, p = 0,54.

To check for enrichment of H3.3 containing nucleosomes, we probed primary spermatocyte nuclei for H3.3S31ph (due to lack of an appropriate antibody detecting unmodified H3.3) and H3.1/H3.2. The high signal for H3.3S31ph in the late pachytene XY body showed that nucleosomes containing H3.1/3.2 were indeed replaced by nucleosomes containing H3.3 (Figure 1C). Variation in signal intensity was observed for H3.3S31ph (n = 20, P2, Table S1). However, this showed no relation to the loss of H3.1/3.2 (not shown).

To conclude, we observed a considerable variation in gonosomal chromatin remodeling during pachytene, both within and between probands, contrasting strongly with our previous observations in mice [22].

### No H3.1/3.2 nucleosome replacement at Yq heterochromatin

**Figure 2:**
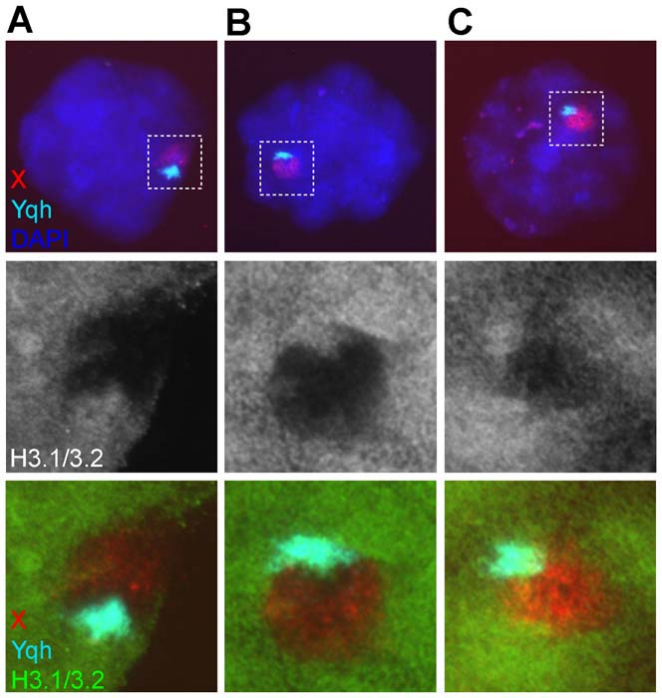
Differential H3.1/3.2 nucleosome remodelling between Yq heterochromatin and the X chromosome in late pachytene spermatocytes. **A** Late pachytene nucleus showing a high degree of nucleosome remodelling for the X chromosome, but no remodelling for Yqh. **B** Late pachytene nucleus showing an intermediate degree of nucleosome remodelling for the X chromosome, but no remodelling for Yqh. **C** Late pachytene nucleus showing a low degree of nucleosome remodelling for the X chromosome, but no remodelling for the Yqh.

In about 50% of the XY bodies with the highest H3.1/H3.2 nucleosome replacement (n = 41, patients and controls, Table 1), we noticed a protrusion of H3.1/3.2 signal, probably of sex chromatin origin (Figure 1B). An axial element loop emanating from the SYCP3-stained tangled knot reached towards or into this protrusion. The loop was observed in 65% of all late pachytene spermatocytes (n = 326, Table 1, patients: 62%; controls: 70%). We performed FISH with an X chromosome painting probe and a Yq heterochromatin (Yqh) probe followed by IF analysis to determine whether there could be a systematic overlap between X and/or Y chromatin signals and H3.1/3.2 signal (Figure 2). From individual C3, 33 late pachytene nuclei were scored of which five showed highest nucleosome eviction. In four of these a clear H3.1/3.2 protrusion was observed, with a co-localizing Yqh probe signal (Figure 2A). In the other 28 nuclei with an intermediate to low degree of H3.1/3.2 nucleosome eviction, overlapping signals were difficult to analyse because of remaining H3.1/3.2 nucleosomes in the XY body. However, Yqh signal was always found together with H3.1/3.2 signal (Figure 2B, C). In some of these nuclei a protrusion could be observed as well (Figure 2B). As expected, the X chromosome showed variable H3.1/3.2 nucleosome eviction (Figure 2A–C). Overall, in nuclei with highest nucleosome eviction, H3.1/3.2 signal was only left on Yqh and at the edges of the DAPI bright domain. H3.3S31ph signal was not enriched on the H3.1/3.2 protrusion and therefore confirmed this notion (Figure 1C). In nuclei with lower nucleosome eviction, H3.1/3.2 depletion was in most cases clearest in the centre of the XY chromatin area where the X probe signal was located (Figure 2C).

### H3.1/3.2 nucleosome replacement and H2AX phosphorylation at S139

**Figure 3:**
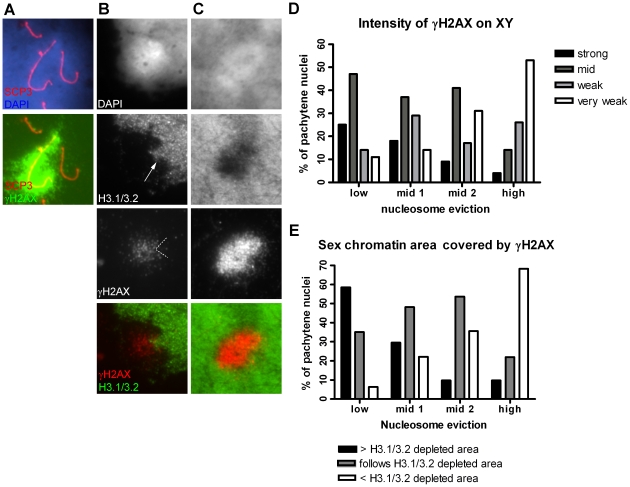
γH2AX in early and late pachytene XY bodies. **A** Early pachytene nucleus. The complete X and Y chromatin is covered by γH2AX. **B** Late pachytene XY body with a high degree of H3.1/3.2 nucleosome remodelling and a decreased γH2AX signal (very weak, < H3.1/3.2 depleted area). A clear bulge of H3.1/3.2 signal is present (arrow) with almost no overlaying γH2AX signal. H3.1/3.2 bulge indicated by the dotted lines. **C** Late pachytene XY body with a low degree of H3.1/3.2 nucleosome remodelling and a strong γH2AX signal (> H3.1/3.2 depleted area). **D** Histogram displaying the relation between the degree of H3.1/3.2 nucleosome remodelling and the intensity of the γH2AX signal, classified into four categories (bars) (n = 203, P1-3, C3). **E** Histogram displaying the relation between the degree of H3.1/3.2 nucleosome remodelling and the area of XY chromatin covered by γH2AX, classified into four categories (bars) (n = 203, P1-3, C3).

γH2AX is a marker of sex chromatin during pachytene, indicating chromatin remodelling leading to meiotic sex chromosome inactivation (MSCI) [5]. We were interested to study whether there is a relation between the degree of sex chromatin nucleosome eviction and the intensity of the γH2AX signal. In early and mid pachytene a strong γH2AX signal was consistently found in the XY body (n = 74, Figure 3A). In the majority of late pachytene nuclei with low XY nucleosome eviction, a strong to mid γH2AX signal remained, extending into H3.1/3.2 positive sex chromatin (Figure 3C,D,E). In nuclei with higher nucleosome eviction the γH2AX signal became weaker (Figure 3B,D). Also the area of XY chromatin which was stained by γH2AX became smaller and usually was in the centre of the XY body (Figure 3B,E). When a H3.1/3.2 Yq heterochromatin protrusion was identified in highly remodelled XY bodies (44%, n = 41) the overlaying γH2AX signal was often weaker (72%, n = 18, Figure 3B). Complete absence of the γH2AX signal was never observed in late pachytene and as human meiosis has no significant diplotene stage [25], [41], [42] we investigated metaphase I nuclei. This is a rare stage in spreading preparations of human spermatogenic cell samples as well. In those found, we did not observe any γH2AX signal (n = 4, not shown). To conclude, in human late pachytene nuclei the disappearance of γH2AX is related to the degree of nucleosome eviction, which constitutes another difference from mice, in which the sex bivalent is characterized by heavy γH2AX staining up to the diplotene stage [4].

### Meiotic sex chromosome silencing

**Figure 4:**
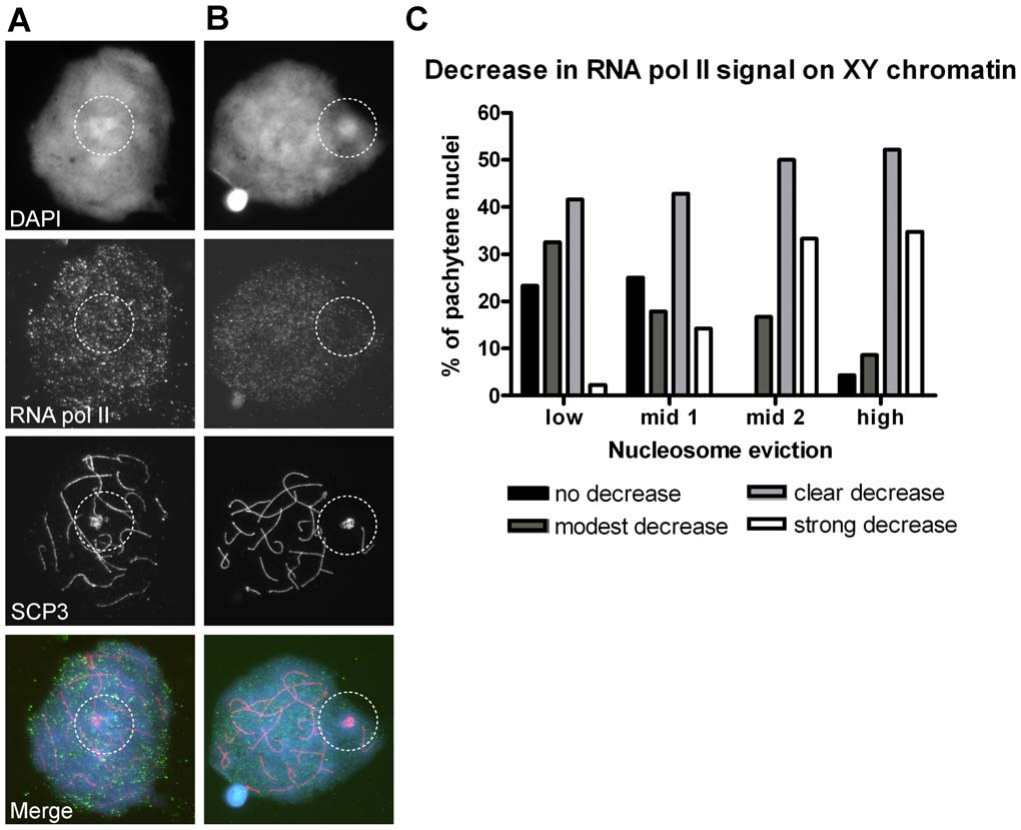
Variation in presence of RNA polymerase II in late pachytene XY bodies. **A** Late pachytene nucleus, showing no decrease in RNA pol II signal intensity at the XY body compared to the autosomal chromatin. Nuclei were stained with the mouse monoclonal antibody. **B** Late pachytene nucleus, showing a strong decrease in RNA pol II signal intensity at the XY body compared to the autosomal chromatin. Nuclei were stained with the mouse monoclonal antibody. **C** Histogram displaying the relation between the degree of nucleosome eviction and the decrease of RNA pol II signal at the XY body, classified into four categories (bars). Total absence of RNA Pol II signal was never observed. Nuclei were stained with the rabbit polyclonal antibody (n = 106, P2, C1-3, Table S1).

In the mouse, we suggested H3.1/3.2 nucleosome replacement to be functional in MSCI [22]. If this supposition is right, then MSCI would constitute a much more variable phenomenon in the human. To investigate MSCI in man, we first stained patient and control nuclei for SYCP3 or H3.1/3.2 in combination with RNA polymerase II (RNA pol II), an indirect marker of transcription. In mouse it was shown that RNA pol II is largely absent at sex chromatin of late pachytene nuclei, while it is strongly present on autosomal bivalents [43], [44]. Our results on human primary spermatocytes, illustrated and graphically represented in Figure 4, show variation in the intensity/presence of the RNA pol II signal on the XY body in late pachytene spermatocytes (compare Figure 4A,B) and suggest a relation between the degree of H3.1/3.2 nucleosome eviction and the amount of RNA pol II demonstrable (Figure 4C). Nuclei with low nucleosome replacement show more RNA pol II than nuclei with high nucleosome replacement. However, in nuclei with high nucleosome eviction we never saw complete absence of RNA pol II signal. Notably, in nuclei with high H3.1/3.2 nucleosome loss no distinction in RNA pol II signal intensity was observed between Yqh (the H3.1/3.2 protrusion) and remaining XY chromatin.

Second, we performed RNA FISH using Cot-1 DNA as a probe. The Cot-1 probe is used to visualize repetitive sequences in intronic and 3′ untranslated regions which are present throughout the genome and emerge upon gene transcription into pre-spliced RNA [8]. To demonstrate genomic coverage, we performed DNA FISH with the Cot-1 probe on human lymphocyte metaphase nuclei and pachytene spermatocytes. In both types of nuclei the complete chromatin, including X and Y, was covered with Cot-1 DNA signal (Figure S2A,C), the signal being stronger for constitutive centric heterochromatin.

**Figure 5:**
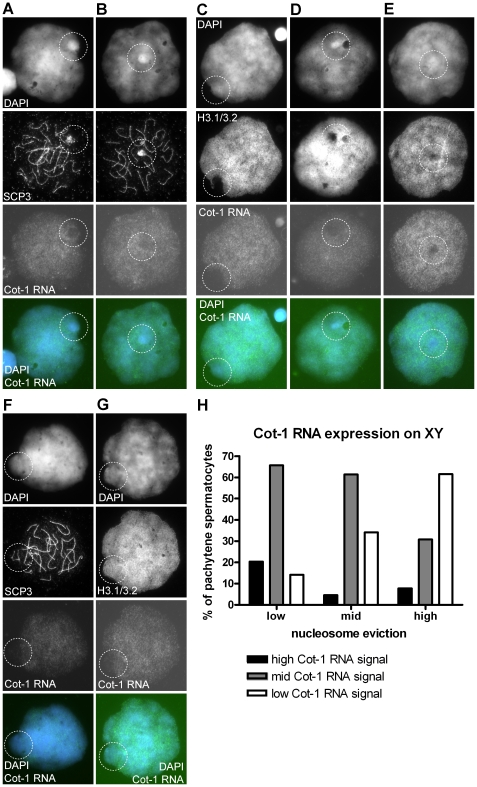
Cot-1 RNA expression in pachytene spermatocytes. A Late pachytene nucleus displaying a low Cot-1 RNA signal on XY. **B** Late pachytene nucleus displaying a higher Cot-1 RNA signal on XY.**C** Late pachytene nucleus showing a high degree of H3.1/3.2 nucleosome eviction and a low Cot-1 RNA signal on XY. **D** Late pachytene nucleus showing an intermediate (mid) degree of H3.1/3.2 nucleosome eviction and intermediate (mid) Cot-1 RNA signal on XY. **E** Late pachytene nucleus showing a low degree of nucleosome eviction and high Cot-1 RNA signal on XY. **F** Early pachytene nucleus displaying low Cot-1 RNA expression on XY. **G** Early or mid pachytene nucleus before H3.1/3.2 nucleosome eviction but with an intermediate (mid) Cot-1 RNA signal. **H** Histogram showing the relation between the degree of H3.1/3.2 nucleosome eviction and the level of Cot-1 RNA signal (bars) (n = 134, P2, C2,3).

To detect transcripts in the XY body, we combined RNA FISH with IF for SYCP3 in P2,3 and C3 (see Table S1). Sertoli cell nuclei were the most heavily stained, corresponding to their active state, while at meiotic metaphase no signal was present (not shown). As expected, very weak RNA signals were seen in the early meiotic prophase stages (leptotene, zygotene). In early pachytene nuclei, the signal intensity increases for the autosomal bivalents (not shown). Over the late pachytene XY bodies of all three men, there was a clear reduction of Cot-1 RNA signal, contrasting to the autosomes. However, variation was observed (Figure 5A: low; B: high). Of all late pachytene nuclei scored (n = 117), 4% showed high Cot-1 RNA staining over the XY body, 54% an intermediate (mid) staining and 42% a low Cot-1 RNA signal. In all early, mid pachytene nuclei in which the XY axial elements were well recognizable, a decrease in Cot-1 RNA was observed (n = 38, Figure 5F).

To link H3.1/3.2 nucleosome eviction to the presumptive silencing of XY genes, we combined RNA FISH with IF for H3.1/3.2 in late pachytene nuclei (n = 134, P2 and C1,3, Table S1). Nuclei showing low nucleosome eviction displayed more Cot-1 RNA and *vice versa*, nuclei with the highest nucleosome eviction showed less Cot-1 RNA (Figure 5C,D,E,H). In nuclei with high H3.1/3.2 nucleosome loss, no distinction in Cot-1 signal intensity was observed between the H3.1/3.2 protrusion (Yqh) and remaining XY chromatin. In agreement with the observation of a decrease of sex chromosome Cot-1 signal in early to mid pachytene, nuclei were found that did not show any loss of H3.1/3.2 staining but did show a local depression of Cot-1 RNA probe hybridization (Figure 5G).

**Figure 6:**
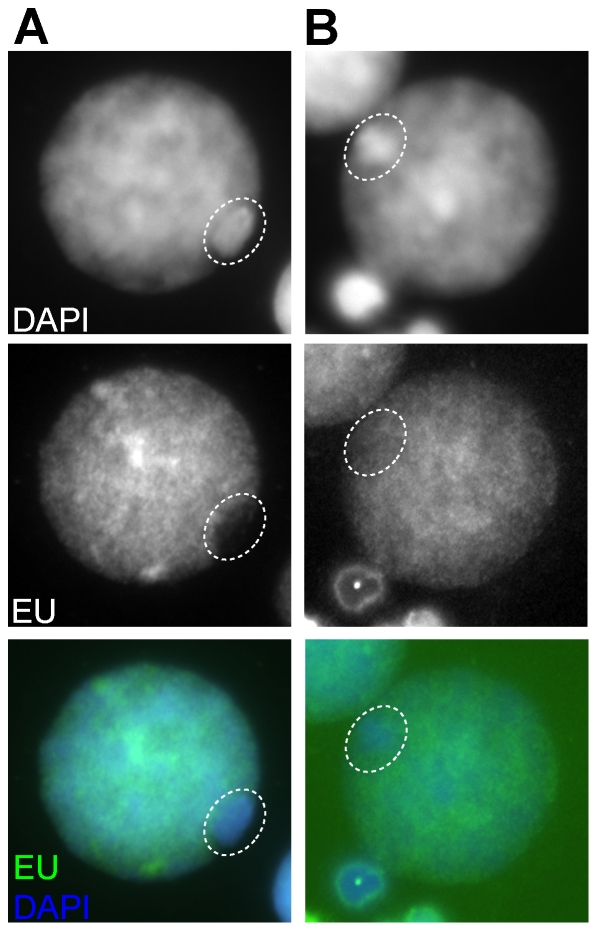
Variation in transcriptional activity of the XY body determined by EU incorporation. The DAPI intense XY body is indicated by an oval in both late pachytene nuclei. **A** Low RNA expression at the XY body. **B** High RNA expression at the XY body.

Third, we cultured spermatogenic cells derived from a fresh TESE biopsy (P4, Table S1, Materials and Methods) with the uridine analog EU. Pilot experiments indicated acid fixation and spreading to better preserve the RNA signal than did basic (PFA) fixation and spreading. Identification of late pachytene nuclei could still be based on the prominent DAPI intensive XY body (Figure 6A,B). Consistent with the results from Cot-1 RNA FISH, Sertoli cells displayed the strongest EU signal and meiotic metaphase nuclei displayed no signal (not shown). In meiotic nuclei without a DAPI intense XY body, autosomal EU signals were low (not shown). In nuclei with an XY body, the autosomal signal was, in most cases, stronger. Late pachytene nuclei were variable in RNA transcriptional activity over the XY body (Figure 6, compare A with B). About 5% of nuclei showed a relatively high production of RNA on the XY body (Figure 6B), 42% an intermediate (mid) expression and 53% of the nuclei displayed only low RNA expression (Figure 6A) (n = 60, P4, Table S1). Complete absence of EU signal was never observed. Taken together, both indirect (by RNA Pol II IF) and direct demonstrations of transcription (by Cot1 RNA FISH and EU incorporation) do show MSCI to be variable between human primary spermatocytes. Double labelling for H3.1/3.2 showed a correlation between higher gonosomal transcription and lower nucleosome remodelling, supporting our original supposition of a mechanistic link between this phenomenon and MSCI [22].

### Transmission of incompletely H3.1/3.2-evicted sex chromosomes to the round spermatid stage

**Figure 7:**
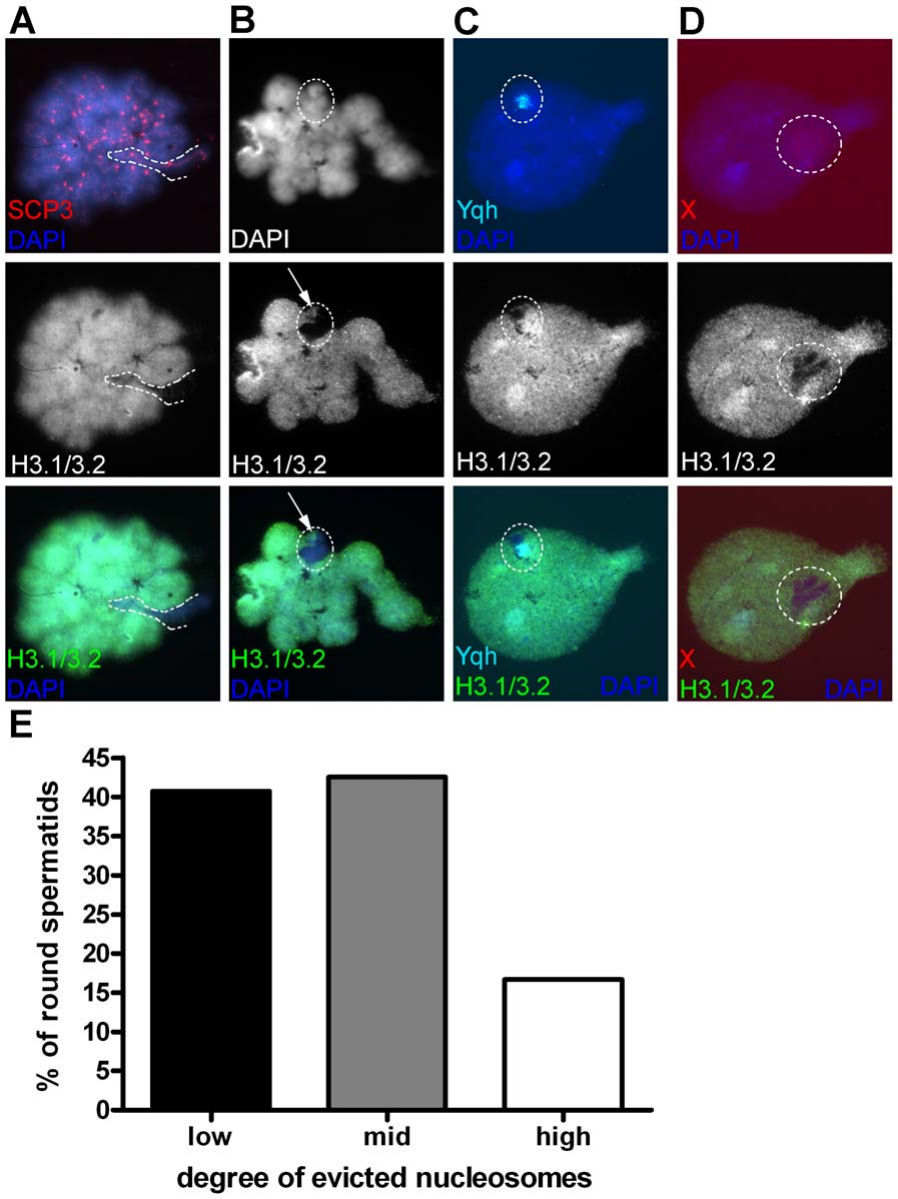
Transmission of incompletely evicted sex chromatin to round spermatids. **A** Metaphase I spermatocyte with a high degree of XY bivalent H3.1/3.2 nucleosome eviction. A bulge of H3.1/3.2 (arrow) was present in a more intense DAPI domain that is part of the encircled sex bivalent, i.e. Yqh. **B** Metaphase I spermatocyte with an intermediate degree of XY bivalent H3.1/3.2 nucleosome eviction (the area within the dashed line). **C** Male early round spermatid nucleus. The Yqh signal colocalizes with H3.1/3.2 remaining histones, adjacent to loss of H3.1/3.2 for Y euchromatin. **D** Female early round spermatid nucleus. Loss of H3.1/3.2 containing nucleosomes was incomplete (mid class) over the X chromosome. **E** Histogram showing the degree of H3.1/3.2 nucleosome eviction for the X chromosome in female early round spermatids (n = 54, C3).

In the mouse, disturbances in MSCI have been associated with the pachytene checkpoint and spermatocyte death (see introduction). Therefore we were interested to discover if at the meiotic divisions there has been selection for spermatocytes with more fully remodelled XY bodies to reach the round spermatid stage. Therefore, we first studied meiotic metaphase I/II nuclei, of which we found 32. In these, some gonosomal H3.1/3.2 remnants were always observed, concordant with the varying degree of replacement found in late pachytene (Figure 7B). The best XY remodelled nuclei showed a little cloud of H3.1/3.2 on a more DAPI intense sex chromatin domain, reminiscent of the absence of nucleosome eviction for Yqh (Figure 7A). These results suggest that incomplete XY H3.1/3.2 replacement does not influence the efficiency of the meiotic divisions, as it does in mice. Consequently, round spermatids with incompletely remodelled sex chromatin must be present.

We utilized IF/FISH analysis (X chromosome painting probe, Yqh probe combined with IF staining of H3.1/3.2) on proband C3 to analyze round spermatids, that were identified by nuclear morphology and by the presence of only one FISH signal (X or Y, Figure 7C,D). Yqh FISH confirmed the lack of nucleosome replacement for this region (Figure 7C). The X chromosome painting probe allowed the study of nucleosome eviction over the whole chromosome (n = 54) (Figure 7D,E): In 40% of spermatid nuclei, the degree of evicted H3.1/3.2 containing nucleosomes was low. In 43% of nuclei there was an intermediate degree of H3.1/3.2 nucleosome eviction (example in Figure 7D) and in the remaining nuclei (17%) the degree of nucleosome eviction was high. These findings were compatible with the interpretation of nucleosome eviction in late pachytene spermatocytes of this proband (Table 1) In all, the data support the concept that meiocytes metamorphose into spermatids irrespective of the level of gonosomal nucleosome eviction.

### Histone characteristics of human meiotic and post-meiotic sex chromatin

**Figure 8:**
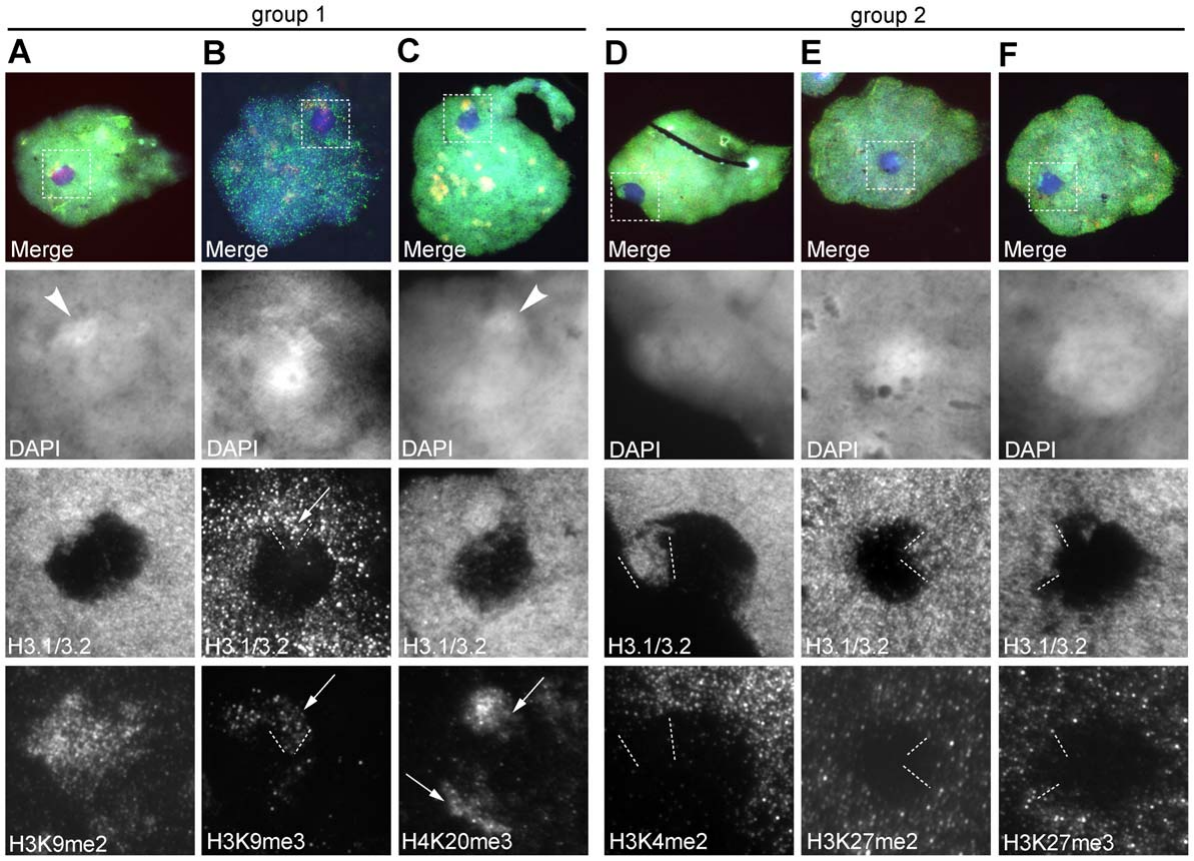
Histone characteristics of the XY body. **A–F** Late pachytene nuclei are shown with a mid-2 to high degree of H3.1/3.2 nucleosome eviction. In the merge panel DAPI, H3.1/3.2 and the PTM are depicted. **A** H3K9me2 was strongly present on XY chromatin including Yqh, as indicated by the brighter DAPI domain (arrowhead). **B** H3K9me3 was present on the Yqh H3.1/3.2 bulge (arrow) and irregular on other XY chromatin. **C** Two H4K20me3 stained domains were found in and associated with X,Y chromatin and were co-staining with H3.1/3.2. Part of the upper domain was located in brighter DAPI XY body chromatin (arrowhead) reminiscent to Yqh. **D–F** H3K4me2, H3K27me2 and H3K27me3 were strongly decreased in X,Y chromatin, including Yqh as indicated by the dotted lines.

Because of the contracted nature and heterochromatic appearance (judged by DAPI staining) of late pachytene spermatocyte XY bodies in the human and mouse and the prominent presence of H3K9me3 in late pachytene XY bodies in the mouse [22], we studied several histone modifications in combination with H3.1/H3.2 staining (Figure 8, Table 2, Figure S3). For both H3K9me3 and H3K4me2 detection, two independent antibodies (see Materials and Methods) were used which did not differ in staining pattern (data not shown). Based on our results and on IF staining patterns that are commonly observed in mouse [9], [22], the histone 3 N-tail methyl PTMs tested for could be divided in two groups: 1) H3K9me2,3 and H4K20me3, which on autosomes at late pachytene yielded a heterochromatin staining pattern (determined by co-staining with the centromere-specific antiserum CREST, data not shown) (Figure 8 A–C); 2) Histone modifications that showed an even autosomal staining, such as H3K4me2 and H3K27me2,3. The latter marks disappeared from the sex chromatin during nucleosome eviction and were also lost from Yqh while H3.1/3.2 was still present (Figure 8D–F, Table 2, Figure S3C–E).

**Table 2:** Histone variant- and histone N-tail-modification dynamics at meiotic and post-meiotic X,Y chromatin.

Histone modification	General dynamics on X,Y	Appearance on Yqh	Early round spermatids
H3K9me2^1^	increase? (n = 176)	present	+/− or + (n = 82)
H3K9me3^1^	decrease, domains (n = 146)	present	− or +/−, domains (n = 95)
H4K20me3^1^	decrease, domains (n = 115)	present	− or +/−, domains(n = 115)
H3K27me2^2^	disappears (n = 182)	disappears	**−** (n = 107)
H3K27me3^2^	strong decrease (n = 171)	strong decrease	− or +/− (n = 106)
H3K4me2^2^	disappears (n = 75)	disappears	− or +/− (n = 27)

Histone variant modification			
H3.3S31ph	strongly present (n = 10)	present	+ (n = 17)
γH2AX	decreases (n = 203)	decreases	− *

1 group 1: histone lysine methyl modifications displaying autosomal heterochromatin staining.

2 group 2: histone lysine methyl modifications displaying autosomal even staining.

In parenthesis: number of nuclei.

*No quantitative analysis was performed on γH2AX staining in early round spermatids as this modification was never observed.

Modifications were studied in P1-3 and C3, except for H3K4me2 which was studied in P2 and C3, and H3.3S31ph which was studied in P2.

H3K9me3 decreased over the XY body in conjuncture with H3.1/H3.2 loss as well, but more selectively as often clearly stained domains remained (Figure 8B; Table 2, Figure S3B). An identical pattern was observed for H4K20me3 (Figure 8C). These domains (for H4K20me3 often more than one per XY body) were located in the periphery of the DAPI-intense sex chromatin. When a H3.1/3.2 protrusion (i.e. Yqh) was present (n = 37), colocalization with H4K20me3 was observed in 95% of cases (Figure 8C). In only 12% of the H4K20me3-stained nuclei (n = 115) the signal was absent from the XY body. Similarly, H3K9me3 was present in all observed Yqh protrusions (Figure 8B) (11%, n = 146) and in seven cases exclusively so.

The staining pattern of H3K9me2 differed from the H3K9me3 and H4K20me3 heterochromatin markers, as no specific domains were associated with the XY body. We observed three types of H3K9me2 staining patterns in late pachytene spermatocytes (Figure S3A) without a clear link to H3.1/3.2 nucleosome eviction. However, a trend is visible towards more H3K9me2 staining on XY in higher H3.1/3.2 remodelled nuclei (Figure S3A, Table 2).

In early round spermatids (Table 2), the sex chromosome signals for histone modifications resembled those observed in late pachytene XY bodies. Least variability was found for modifications that tended to be low at XY during/after nucleosome eviction such as H3K4me2, H3K27me2 and H3K27me3. H3K9me2 reached higher coverage over the sex chromosomes than did H3K9me3, which was faintly present and showed stronger domains next to the H3.1/3.2-evicted PMSC, as in late pachytene. In the majority of spermatid nuclei H4K20me3 seemed to be faintly present over the X or Y, while this was not observed at late pachytene. Furthermore, similar domains were associated with the PMSC as found for H3K9me3 staining. In most cases H3.3S31ph was stronger on the sex chromatin compared to autosomal chromatin. We never observed γH2AX in round spermatids.

In conclusion, with the exception of Yqh, the sex chromosomes at late pachytene miss an overt heterochromatic signature as based on H3 and H4 N-tail modifications. In early round spermatids, this pattern is maintained with the addition of H4K20me3 that might signal heterochromatinization.

## Discussion

### MSCI in the human male

In the present study we show by indirect and direct cytological methods (H3.1/3.2 nucleosome eviction and presence of RNA polymerase II, EU incorporation and Cot-1 RNA FISH) MSCI to occur in man. In late pachytene, H3.1/3.2 nucleosome eviction is correlated with the repression of Cot-1 RNA expression. Using the loss of H3.1/3.2 as an indirect marker for MSCI, variation was detected between and within patients and controls. Cot I RNA FISH and EU incorporation confirmed this observation. This variability did not occur for H3.1/3.2 nucleosome eviction in spermatocytes from Swiss random bred mice [22]. To follow up and confirm this observation, we checked H3.1/3.2 nucleosome eviction in spermatocytes from a Swiss derived outbred NMRI mouse and a Swiss derived inbred FVB mouse. Similarly, both mice had homogeneously H3.1/3.2 evicted late pachytene XY bodies that by this characteristic usually were sharply demarcated (data not shown). Variation in MSCI has neither been specifically noticed in ^3^H-uridine uptake studies using inbred C3H mice and random bred Swiss mice [6], [30].

In the human, variation was most apparent at the circumference of the XY body that is much more fuzzy, as observed by DAPI staining, compared to mouse. Also stainings by H3,H4 PTMs that mark heterochromatin failed to uniformly label gonosomal chromatin at late first meiotic prophase, with the exception of Yqh. Our notion that nucleosome remodelling is linked to MSCI is strengthened by its absence in Yqh. Apparently, this domain is sufficiently made incapable of mechanistic transcription by its intrinsic chromatin structure, illustrated by the histone PTM stainings. In the mouse, pachytene XY body localization is related to the pachytene substage [45]. The later in pachytene, and clearly from stage IV on, the more the XY body was found in the periphery of the nucleus. For chromatin remodelling, we studied late pachytene spermatocyte XY bodies only, which according to the available mouse data [45] should have a preference for the nuclear periphery. However, we found XY bodies to be located over the whole spread area. Low H3.1/3.2 remodelled late pachytene XY bodies tended to be located more towards the centre compared to highly remodelled late pachytene XY bodies that were more often found near the periphery. One explanation for this phenomenon could be that for maintenance of MSCI a peripheral localization is advantageous.

In the mouse, MSCI commences at early pachytene [6]. Cot-1 RNA FISH in combination with staining for SYCP3 revealed that from early to late pachytene, Cot-1 RNA was reduced at the human XY body. The first sign of gonosomal H3.1/3.2 loss in human spermatocytes was at mid pachytene, which is different from the mouse, where H3.1/3.2 nucleosome eviction starts at early pachytene [22]. These results indicate that also in the human, H3.1/3.2 nucleosome eviction follows X,Y gene silencing, though with a greater time lapse.

The general idea is that MSCI is initiated by the cascade of events following H2AX S139 phosphorylation [5] the spreading of which is assisted by MDC1 [19]. Strong γH2AX staining is present in both human and mouse early pachytene spermatocytes, indicating that initiation of MSCI is similar in human and mouse. This is reflected by an inactive XY body in early pachytene for both human (this study) and mouse [6]. The variation in MSCI we observed in late pachytene might therefore be caused by variation in the maintenance of silencing in which H3.1/3.2 nucleosome remodelling (and resulting histone PTM changes) might have a role, concomitant with the chromatin domain moving away from the transcriptionally active autosomal bivalents.

Could the decrease in γH2AX staining we observed in human late pachytene be involved in maintenance variation of MSCI? In the mouse, staining continues into diplotene and is lost at first meiotic metaphase, which is different in the human (who are missing a substantial diplotene stage [25], [41], [42]). Here this mark is reduced up to a very low level when nucleosome remodelling is maximal (late pachytene). For the mouse, an interpretation could be that this modification helps in maintaining MSCI. In the human our results do not support this view, as the human XY bodies showing low H2AX S139 phosphorylation were better nucleosome remodelled and on average showed a lower Cot-1 RNA signal.

### No selection for incomplete H3.1/3.2 evicted pachytene spermatocytes

In mouse chromosome mutants in which the association of asynapsed chromosome segments with the XY body interferes with MSCI, pachytene and metaphase I death usually follows [10], [28]. Assuming that this typical pattern of spermatocyte death is related to expression of gonosomal genes, one could ask if such a selection step does occur in human spermatogenesis. In the light of the variation in MSCI indicated by our data, this would not seem likely. We observed variation in X chromosomal H3.1/3.2 depletion in round spermatids, reproducing the variegated pattern found in late pachytene. Also around the Yqh, variation in an adjacent H3.1/3.2 depleted area was witnessed. Hence, there does not seem to be a clear selection against primary spermatocytes with respect to chromatin remodelling, i.e. the global transcriptional status of the sex chromosomes. Observations on XY chromatin remodelling at first and second meiotic metaphase on average yielded the impression of a more fully executed remodelling process, but traces of H3.1/3.2 outside the Yqh area were found. The more fully remodelled impression of the metaphase sex chromosomes may well be caused by the more compacted nature of the chromatin in which fainter histone signals are not picked up. To conclude, these data indicate that in man a variation in meiotic gonosomal expression pattern is tolerated before, and likely also after the meiotic divisions.

Also in the human, the aggregation of unsynapsed autosomal chromatin with the sex chromosomes intervenes with spermatogenesis [31]. As in mouse this increases heterogeneity of human sperm even further and generally results in the oligo-asteno-teratozoospermia (OAT) syndrome as well [46], suggesting that a link between X,Y gene activity and spermatogenic cell death/spermatid differentiation is maintained [24], [47]. Transcriptional inactivation at the unsynapsed autosomal segments might be a contributing factor [47], which is omitted when studying mouse and human males with an XYY karyotype. In the mouse, Y activity during pachytene is detrimental as evidenced by strong selection during first prophase against spermatocytes with a Y bivalent that escapes MSCI [12]. This selection is less strong in the human XYY spermatocytes as is demonstrated in patients in which the habitual loss of one Y chromosome during the spermatogonial divisions did not occur and the Y chromosomes could partly or wholly synapse [48]–[51]. Loss could occur at the meiotic divisions or during spermiogenesis, and to a much lesser extend at pachytene. These observations support our interpretation that Y (and X) expression during first meiotic prophase in the human is less strictly controlled as compared to mouse, leading to the greater between gamete variation as observed in our species [37], [38].

### An evolutionary explanation for variable MSCI in the human

In human, the phenotypes of the AZFb and c deletions allow an indirect genetic analysis into Y gene activity during spermatogenesis. AZFb presents with a uniform early meiotic prophase maturation arrest [52] suggesting transcription from the Y being required before MSCI. In the AZFc deletion hypospermatogenesis is found, leading to ejaculated sperm in 81% of men [53], [54] suggesting that absence of AZFc gene transcripts leads to variation in cell fate. Table S2 lists several genes from the male specific region of the Y (MSY), mostly of the AZFa, b and c regions, from which only scarce *in situ* gene expression data (RNA and protein) were available in the literature. Although gene specific RNA FISH is needed to confirm the pattern presented in this table, the suggestion is that some genes do display MSCI (DAZ, SRY, TSPY) while others do not (DDX3Y, RBMY, SMCY, RPS4Y). DDX3Y and RBMY are also present at the mouse Y where they are subjected to MSCI [12].

When we compare the MSY gene content in human [55] and mouse [12], several more differences can be observed: first, the number of protein-coding genes is lower in mouse (15) (human: 27) and second, only 40% of the mouse Y genes are preserved on the human Y illustrating the high rate of evolution, despite the fact that the Y specialises in spermatogenesis in both species. The recent finding of remarkable differences in MSY gene content between human and chimpanzee stresses the rate of evolutionary change even further [56]. These authors observed that compared with man, the chimpanzee Y chromosome had lost 4 out of 16 genes from the X degenerate regions that do not have a spermatogenesis specific function. Interestingly, a recent *in silico* analysis on array hybridisation expression data of whole testes from the chimpanzee and man revealed signs of MSCI for the chimpanzee but could not demonstrate decreased expression of human XY genes [26].

Among the evolutionary forces that shape the Y chromosome, as summarized by Hughes [56], there is first of all specialization of gene function towards spermatogenesis, secondly the high frequency of intrachromosomal ectopic recombination, and thirdly the absence of homologous interchromosomal recombination (for the MSY region). Furthermore, sperm competition within the female reproductive tract likely speeds up selection and hence evolution of the Y chromosome [56]. From human population genetics [57]–[59] and physiological data [60] it can be deduced that in the human polygyny has not been the norm over our recent evolutionary history though it likely prevailed over earlier times. Hence, multifemale-multimale mating systems are assumed to be rare [60]. This may have caused a relaxation of MSCI. This relaxation might also be demonstrated by the fact that some AZFb, c genes probably are subjected to MSCI and others not (Table S2). Thereby, systematic MSCI is alleviated to allow Y gene activity in pachytene and most likely the post meiotic stages. As a by product, the heterochromatinization of the meiotic sex chromosomes in late pachytene as witnessed in mouse is much less pronounced in man. Also, the selective pressure on morphological uniformity in gamete quality, that seems better guarded by complete MSCI and is here assumed to be functional in sperm competition between males, has been given up. Relaxation of MSCI might also involve a difference in activation of the pachytene checkpoint which in mouse was found to be induced by miss expression of Zfy1/2 [12]. When MSCI is less effective in human, human ZFY transcripts might not activate this checkpoint, as also is indicated by the occurrence of meiotic divisions in XYY men, who escape MSCI when a Y bivalent is formed [51]. However, of the ZFY/ZFX gene family which is present in both mouse and man, mouse *Zfy1/2* are the least conserved, enabling evolution of function [61]. Alternatively, the human pachytene checkpoint might be placed before the time of relaxation of MSCI. However, this theory would not hold for XYY spermatocytes with a Y bivalent. Our observations need follow up by determining gonosomal gene expression in the spermatocytes and round spermatids, especially as also in the mouse, MSCI is not systematically executed: The majority of X-linked miRNAs (86%) escapes MSCI [62].

Conclusively, we postulate variation in meiotic and post-meiotic X,Y chromatin repression to contribute to morphological sperm heterogeneity, the consequences of which are alleviated by alternative selection mechanisms like within proband sperm selection and early embryonic death [63], which does not lead to a reproductive disadvantage for the couple.

## Materials and Methods

### Ethics Statement

On October 18^th^, 2006, the CCMO (Central Committee on Research involving Human Subjects) approved of the research protocol entitled ‘*Intracytoplasmatic Sperm Injection using testicular spermatozoa in men with azoospermia: an observational study’*.

### Human testis material

Testis material was obtained from seven men (patients and controls) with non-obstructive azoospermia (NOA) or obstructive azoospermia (OA) willing to conceive, who underwent a testicular biopsy for sperm retrieval (TESE: testicular sperm extraction). One large biopsy was taken following the procedure of Silber [64]. From all probands a drop of spermatogenic cell suspension was smeared on a microscope slide prior to sperm retrieval. Cells were Giemsa stained and pachytene spermatocytes, spermatozoa and Sertoli cells were counted. In Table S1, ratios between pachytene nuclei and mature elongated spermatids (sperm) and between mature elongated spermatids and Sertoli cells are given. The latter ratio indicates the spermatogenic activity of the tissue sampled, the former ratio the efficiency of the production of mature spermatids per meiotic cell. Although not strictly comparable with histological studies, our data are in line with those published [65]–[67]. Spermatogenic cells were available for research, after sperm retrieval had been successful. Patients were selected that at first inspection had relatively high numbers of spermatozoa in the biopsy. The diagnosis of NOA was established after repeated absence of spermatozoa in the ejaculate in combination with an elevated FSH level (>15 IU/ml) and no indications for an obstruction (Table S1). In cases of OA, repeatedly no spermatozoa were found in combination with a medical history of obstruction and/or normal FSH levels (Table S1). Testis material from men of proven fertility, with a history of a vasectomy and absence of spermatozoa in the caput epididymis thereafter (OA), was used as control material (C1/2/3 Table S1). Vasovasostomy testis material is often used to represent normal spermatogenesis [25], [68]–[70]. For control men 1 and 2, histology of the biopsy was evaluated by the Johnsen score [71]. Normal spermatogenesis was indicated by scores of 9.1 and 9.5 (with tubule scores of only 9 and 10). All men signed an informed consent for participation in this project. Testis material was used from one outbred NMRI and one inbred FVB mouse, both Swiss random bred derived. The procedure involving these animals is approved by the animal ethics committees of the Radboud University Nijmegen Medical Centre and Wageningen University and Research Centre, in conformance with the Dutch law on the use of experimental animals.

### Surface spread preparations

Nucleus spreads were made as described by Peters [72], with minor modifications. Briefly, a suspension of spermatogenic cells was made by crushing the remnant seminiferous tubuli with two ribbed forceps in a drop of SIM (spermatocyte isolation medium [73]). Remnants were separated from the cell suspension by a quick spin (25 g). The supernatant was transferred to a clean tube, centrifuged for 7 minutes (159 g), and the pellet was resuspended in 1 ml SIM. An equal volume of a hypotonic solution (17 mM sodium citrate, 50 mM sucrose, 30 mM Tris-HCl pH 8.2) was added for 7 minutes. Cells were centrifuged again (7 minutes, 159 g) and resuspended in 100 mM sucrose (pH 8.2) at a concentration of 10–15×10^6^/ml. Two 5 µl drops were pipetted onto a paraformaldehyde (1% PFA, 0.15% Triton-X-100 pH 9.2) fluid coated microscope slide, placed in a levelled humid box for about 75 minutes, rinsed twice in 0.08% photoflow (Kodak), and air dried. Slides were stored at −80° until use.

### Immunofluorescence (IF)

Surface spread preparations were washed twice in PBS containing 0.05% Triton-X-100 and blocked for 1 hour at 37°C (blocking buffer: 1% bovine serum albumin, 10% normal goat serum or normal donkey serum in PBS containing 0.05% Triton-X-100). Primary antibodies were diluted in blocking buffer and slides were incubated for 20 minutes at 37°C, followed by overnight incubation at 4°C (when using the RNA polymerase II antibodies, we incubated 2 nights at 4°C) extended by 20 minutes at 37°C. Then slides were rinsed and washed once in PBS containing 0.05% Triton-X-100 and afterwards rinsed and washed once in PBS. A second 30 minute blocking step was applied in blocking buffer without Triton-X-100, followed by a 2 hour incubation with the secondary antibodies diluted in blocking buffer without Triton-X-100. After rinsing and washing once in PBS, nuclei were stained with DAPI (10 min, 0.3 µg/ml in PBS) and mounted with Vectashield (Vector). When performing FISH after IF, nuclei were dehydrated in an 70–80–90–100% ethanol series after the last PBS wash.

### DNA fluorescent in situ hybridization (FISH) with directly labelled probes

FISH analysis was performed with directly labelled whole chromosome X (LPP0X/R Aquarius, Cytocell) and Yq12 (32-131024 CEP Y (satellite III) SpectrumAqua, Abbott Molecular Inc.) probes. DNA was denatured for 3 minutes at 72°C and probes were able to hybridize overnight at 37°C. Slides were washed twice (1 minute) in 0.4× SSC/0.1% Tween-20 at 68°C and subsequently once in 2× SSC, once in 4× SSC and twice in PBS at room temperature. Nuclei were dehydrated in an ethanol series and mounted with Vectashield containing DAPI. For the DNA FISH protocol with biotin labelled Cot-1 probe see Methods S1.

### Cot-1 RNA FISH

Cot-1 RNA FISH was performed as described [8] with modifications to enhance the signal (see Methods S1 and Figure S2A-H for rationales of this RNA FISH protocol). Briefly, human Cot-1 DNA (Invitrogen 15279-011) was labelled with biotin using the BioNick Labeling System (Invitrogen 18247-015). Probes were directly dissolved in hybridization buffer containing 50% formamide (Vysis). Probe was mixed (1∶1) with preheated (37°C) hybridization buffer (4× SSC, 20% dextran sulphate, 2 mg/ml BSA, 2 mM vanadyl ribonucleoside), put onto a surface spread preparation and covered with a 18×18 mm coverslip. Nuclei and probe were heat denatured at 72°C (3 min) and hybridized overnight at 37°C in a humid box. The next day, slides were subjected to several stringency washes specific for RNA FISH starting with 3×5 minutes in 1× SSC/50% formamide at 42°C followed by 3×5 minutes in 2× SSC and subsequently placed in 4× SSC containing 0.1% Tween-20. Preparations were blocked (4× SSC, 0.1% Tween-20, 4 mg/ml BSA) for 30 minutes in a humid box at 37°C. Probe detection was carried out using avidine-FITC followed by goat anti-avidine FITC. Both reagents were incubated for 30 minutes at 37°C. Subsequently, slides were washed in PBS and directly used for primary antibody staining by IF. Primary antibody was diluted in PBS and incubated for 1 hour at 37°C followed by three PBS washes. Secondary antibody was incubated for 30 minutes at 37°C followed by 3 PBS washes. DAPI staining was performed and slides were mounted with Vectashield.

### RNA imaging by 5-ethynyluridine (EU) incorporation

Newly synthesized RNA was visualized by incorporation of the uridine analog 5-ethynyluridine (EU) [74]. Incorporated EU can be detected by a copper-catalyzed cyclo addition reaction with a fluorescent azide (aka ‘click reaction’). From a fresh biopsy (P4, Table S1) a cell suspension was made. After the first centrifuge step (7 minutes, 159 g) cells were resuspended in MEM alpha-complete medium [25]. Small cultures (200 µl) containing about 15×10^6^ cells/ml were made and EU (Invitrogen C10329) was added in a concentration of 4 mM. Cells were cultured for 3 hours at 32°C in an atmosphere containing 5% CO_2_ [25]. Acid-fixated air-dried nuclei were prepared as described by Evans [75]. EU was detected using the Click-iT® RNA imaging kit (Invitrogen C10329) followed by DAPI staining and mounting in Vectashield.

### Antibodies

To localize H3.1/3.2, monoclonal antibody #34 [76] provided by J. van der Vlag was used at a dilution of 1∶1500 for IF, at 1∶1000 before DNA FISH and at 1∶500 after RNA FISH. A rabbit polyclonal antibody against SYCP3 (Abcam, ab15092) was used at 1∶200 for IF and at 1∶50 after Cot-1 RNA FISH. A rabbit polyclonal antibody against γH2AX (Upstate, 07-164) was used at 1∶100. A polyclonal antibody against H3.3 phosphorylated at serine 31 (Abcam, 2889) was used at 1∶100. A rabbit polyclonal antibody against H3K9me2, provided by T. Jenuwein was used at 1∶150. To detect H3K9me3 two rabbit polyclonal antibodies were used, Abcam (ab8898) at 1∶1000, and one generated by A.H.F.M. Peters at 1∶750. Polyclonal rabbit antibodies against H3K27me2,3, provided by T. Jenuwein were used at 1∶350. Two rabbit polyclonal antibody against H3K4me2 were used, Abcam (ab7766) at 1∶100 and Upstate (07-030) at 1∶500. To detect RNA polymerase II we used a rabbit polyclonal antibody (Abcam, ab26721) at a dilution of 1∶20 and a mouse monoclonal antibody (Abcam, ab817) at 1∶50. Centromeres were detected by a human autoantibody (Crest) at a dilution of 1∶500 (ImmunoVision, Springdale AR). Primary antibodies were detected by using goat or donkey anti-mouse and goat or donkey anti-rabbit secondaries with respectively a red or a green fluorochrome at 1∶500 dilution (Invitrogen Alexa 488; A11001, A21202, Alexa 594; A11012, A21207). To detect the biotinylated Cot-1 RNA FISH probe, avidine FITC (Vector, SA-5001) was used in a 1∶500 dilution enhancing the signal with a goat anti-avidine FITC antibody (Vector, SP-2040, 1∶200).

### Analysis and image capture

Staging of pachytene nuclei was based on staining of SYCP3 to visualize the synaptonemal complexes (SCs) [25], in combination with nuclear morphology by DAPI. To determine the degree of H3.1/3.2 nucleosome eviction, the level of Cot-1 RNA hybridisation and the level of EU incorporation of the late pachytene XY body, SYCP3 staining and/or its prominent and intense DAPI signal was used for localization and determination of its area. We subjectively determined the coverage of this area with H3.1/3.2, Cot-1 and EU signal. For H3.1/3.2 staining, late pachytene nuclei were categorized into four groups ranging from low nucleosome eviction to high nucleosome eviction (low > mid1 > mid2 > high) (Figure 1B). Cot-1 (Figure 5A-E) and EU RNA (Figure 6) staining was categorized into three groups (low > mid > high) in which ‘low’ indicates a strong decrease in signal and ‘high’ a modest decrease compared to autosomal staining (see Figures 5, 6). Categorization into three groups instead of four was carried out when fewer nuclei were included in the experiment. For the combination of H3.1/3.2 with Cot-1 RNA FISH, H3.1/3.2-stained late pachytene nuclei were categorized into three groups as well (see Figure 5 C,D,E). To determine the degree of H3.1/3.2 nucleosome eviction in round spermatids, nuclei were categorized into three groups: H3.1/3.2 was reduced on the X chromatin (detected by FISH) but signal was still present (low), H3.1/3.2 was strongly reduced and parts of the chromatin were free of H3.1/3.2 (mid), in a large part of the sex chromatin H3.1/3.2 was completely evicted (high) (Figure 7D).

Staining intensities and patterns of γH2AX, RNA polymerase II and the H3, H4 N-tail PTMs were subjectively determined as well and classified as described in the figure legends. Nuclei were captured by a Zeiss AxioCam MR camera on a Zeiss Axioplan fluorescence microscope using Axiovision 3.1 software (Carl Zeiss). Around 50 late pachytene and 25 round spermatid nuclei were captured per patient/control man per immunostaining at an exposure time reflecting the microscopic image.

### Statistics

Deviations from random class distributions have been tested by Chi square analysis.

## Supporting Information

Figure S1 **XY body localization.** The degree of H3.1/3.2 nucleosome eviction of late pachytene spermatocytes from patients and controls (Table 1) is shown with their accompanying XY body localization. Bars indicate the location of the XY body: in or close to the centre (centre), halfway the centre and the edge (1/2), at two-third from the centre to the edge (2/3), and at the edge (edge). In grey the number of nuclei. Statistical analysis by Chi-square: overall χ^2^: 30.26 df 9 p<0.001, low vs. mid 2 χ^2^: 12.68 df 3, p<0.01, low vs high χ^2^: 24.09 df 3, p<0.001, mid1 vs high χ^2^: 13.35 df 3, p<0.01, low + mid1 vs mid2 + high χ^2^:24.69 df 3, p<0.001.
(EPS)

Figure S2 **Validation of RNA FISH signals at different denaturation temperatures.** **B–H** The dotted circle indicates the XY body. **A** Lymphocyte metaphase. After DNA FISH the Cot-1 probe hybridizes to all chromosomes and showed more intense staining at the centromeric heterochromatin. **B** Pachytene spermatocyte nucleus. After DNA FISH the Cot-1 probe hybridized to all autosomal chromosomes and showed decreased hybridization signal for X,Y chromatin. **C** RNAse-treated pachytene spermatocyte nucleus. After DNA FISH the Cot-1 probe hybridized to all chromosomes with more intense staining at the centromeric heterochromatin. **D** Pachytene spermatocyte nucleus. After RNA FISH at 72°C the Cot-1 probe hybridized to all autosomal chromosomes and showed decreased hybridization signals for XY chromatin. **E** RNAse treated pachytene spermatocyte nucleus. After DNA FISH at 62°C the Cot-1 probe was not able to hybridize. **F–H** Pachytene spermatocyte nucleus. After RNA FISH at 62°C, 52°C and 37°C respectively, the Cot-1 probe hybridized to all autosomal chromosomes and showed decreased hybridization signals for the XY chromatin.
(TIF)

Figure S3 **Graphical representation of histone dynamics at the XY chromatin in late pachytene spermatocytes.** **A–E** Late pachytene nuclei were assigned to four groups, ranging from a low to a high degree of nucleosome eviction (see Figure 1B) (for probands included and number of nuclei see Table 2). Histone characteristics and histone N-tail modifications were subjectively determined for the XY body and in A for autosomal chromatin as well: **A** Nuclei were categorized into three goups (bars). ‘Low staining, higher at XY’ indicates at the observation of low, even autosomal staining with more intense staining at the XY body. ‘General low staining’ indicates at an overall low staining including the XY body. ‘Autosomal domains, strong XY’ points at the observation of a heterochromatin staining pattern on autosomal chromosomes and overall staining of the XY body. **B** The part of the XY chromatin stained was determined and arranged into four groups (bars). **C–E** The decrease in signal for the XY chromatin, compared to the autosomal chromatin, was determined and categorized into classes (bars). Statistical analysis by Chi-square; A: χ^2^ = 21.98 df 6, p<0.01, B: χ^2^ = 47.87 df 9, p<0.001 (Abcam antibody), C: χ^2^ = 29.2 df 6, p<0.001, D: χ^2^ = 40.2 df 9, p<0.001, E: χ^2^ = 10.9 df 6, p = 0.21 (Abcam antibody).
(TIF)

Table S1 **Proband details.**
(DOC)

Table S2 **Literature data on meiotic expression of several MSY/AZFa,b,c genes.**
(DOC)

Methods S1 **Supporting methods.**
(DOC)

## References

[1] Solari AJ (1974). The behavior of the XY pair in mammals.. Int Rev Cytol.

[2] Inagaki A, Schoenmakers S, Baarends WM (2010). DNA double strand break repair, chromosome synapsis and transcriptional silencing in meiosis.. Epigenetics.

[3] Turner JM, Aprelikova O, Xu X, Wang R, Kim S (2004). BRCA1, histone H2AX phosphorylation, and male meiotic sex chromosome inactivation.. Curr Biol.

[4] Mahadevaiah SK, Turner JM, Baudat F, Rogakou EP, de Boer P (2001). Recombinational DNA double-strand breaks in mice precede synapsis.. Nat Genet.

[5] Fernandez-Capetillo O, Mahadevaiah SK, Celeste A, Romanienko PJ, Camerini-Otero RD (2003). H2AX is required for chromatin remodeling and inactivation of sex chromosomes in male mouse meiosis.. Dev Cell.

[6] Monesi V (1965). Synthetic activities during spermatogenesis in the mouse RNA and protein.. Exp Cell Res.

[7] Kierszenbaum AL, Tres LL (1974). Nucleolar and perichromosomal RNA synthesis during meiotic prophase in the mouse testis.. J Cell Biol.

[8] Turner JM, Mahadevaiah SK, Fernandez-Capetillo O, Nussenzweig A, Xu X (2005). Silencing of unsynapsed meiotic chromosomes in the mouse.. Nat Genet.

[9] Namekawa SH, Park PJ, Zhang LF, Shima JE, McCarrey JR (2006). Postmeiotic sex chromatin in the male germline of mice.. Curr Biol.

[10] Homolka D, Ivanek R, Capkova J, Jansa P, Forejt J (2007). Chromosomal rearrangement interferes with meiotic X chromosome inactivation.. Genome Res.

[11] de Rooij DG, de Boer P (2003). Specific arrests of spermatogenesis in genetically modified and mutant mice.. Cytogenet Genome Res.

[12] Royo H, Polikiewicz G, Mahadevaiah SK, Prosser H, Mitchell M (2010). Evidence that meiotic sex chromosome inactivation is essential for male fertility.. Curr Biol.

[13] McKee BD, Handel MA (1993). Sex chromosomes, recombination, and chromatin conformation.. Chromosoma.

[14] Tres LL (1975). Nucleolar RNA synthesis of meiotic prophase spermatocytes in the human testis.. Chromosoma.

[15] Saussine C, Gabriel-Robez O, Rumpler Y (1994). Pattern of ribonucleic acid synthesis in human primary spermatocytes.. Andrologia.

[16] Schoenmakers S, Wassenaar E, Hoogerbrugge JW, Laven JS, Grootegoed JA (2009). Female meiotic sex chromosome inactivation in chicken.. PLoS Genet.

[17] Schimenti J (2005). Synapsis or silence.. Nat Genet.

[18] Sinha M, Peterson CL (2009). Chromatin dynamics during repair of chromosomal DNA double-strand breaks.. Epigenomics.

[19] Ichijima Y, Ichijima M, Lou Z, Nussenzweig A, Camerini-Otero RD (2011). MDC1 directs chromosome-wide silencing of the sex chromosomes in male germ cells.. Genes Dev.

[20] Turner JM (2007). Meiotic sex chromosome inactivation.. Development.

[21] Zamudio NM, Chong S, O'Bryan MK (2008). Epigenetic regulation in male germ cells.. Reproduction.

[22] van der Heijden GW, Derijck AA, Posfai E, Giele M, Pelczar P (2007). Chromosome-wide nucleosome replacement and H3.3 incorporation during mammalian meiotic sex chromosome inactivation.. Nat Genet.

[23] Solari AJ, Tres LL (1967). The ultrastructure of the human sex vesicle.. Chromosoma.

[24] Sciurano R, Rahn M, Rey-Valzacchi G, Solari AJ (2007). The asynaptic chromatin in spermatocytes of translocation carriers contains the histone variant gamma-H2AX and associates with the XY body.. Hum Reprod.

[25] de Boer P, Giele M, Lock MT, de Rooij DG, Giltay J (2004). Kinetics of meiosis in azoospermic males: a joint histological and cytological approach.. Cytogenet Genome Res.

[26] Mulugeta AE, Baarends WM, Gribnau J, Grootegoed JA (2010). Evaluating the relationship between spermatogenic silencing of the X chromosome and evolution of the Y chromosome in chimpanzee and human.. PLoS One.

[27] de Boer P, van der Hoeven FA, Chardon JA (1976). The production, morphology, karyotypes and transport of spermatozoa from tertiary trisomic mice and the consequences for egg fertilization.. J Reprod Fertil.

[28] de Boer P, Searle AG, van der Hoeven FA, de Rooij DG, Beechey CV (1986). Male pachytene pairing in single and double translocation heterozygotes and spermatogenic impairment in the mouse.. Chromosoma.

[29] Turner JM, Mahadevaiah SK, Ellis PJ, Mitchell MJ, Burgoyne PS (2006). Pachytene asynapsis drives meiotic sex chromosome inactivation and leads to substantial postmeiotic repression in spermatids.. Dev Cell.

[30] Speed RM (1986). Abnormal RNA synthesis in sex vesicles of tertiary trisomic male mice.. Chromosoma.

[31] Martin RH (2008). Cytogenetic determinants of male fertility.. Hum Reprod Update.

[32] Baarends WM, Wassenaar E, Hoogerbrugge JW, Schoenmakers S, Sun ZW (2007). Increased phosphorylation and dimethylation of XY body histones in the Hr6b-knockout mouse is associated with derepression of the X chromosome.. J Cell Sci.

[33] Mulugeta AE, Wassenaar E, Hoogerbrugge JW, Sleddens-Linkels E, Ooms M (2010). The ubiquitin-conjugating enzyme HR6B is required for maintenance of X chromosome silencing in mouse spermatocytes and spermatids.. BMC Genomics.

[34] Reynard LN, Turner JM (2009). Increased sex chromosome expression and epigenetic abnormalities in spermatids from male mice with Y chromosome deletions.. J Cell Sci.

[35] Ellis PJ, Clemente EJ, Ball P, Toure A, Ferguson L (2005). Deletions on mouse Yq lead to upregulation of multiple X- and Y-linked transcripts in spermatids.. Hum Mol Genet.

[36] Yamauchi Y, Riel JM, Stoytcheva Z, Burgoyne PS, Ward MA (2010). Deficiency in mouse Y chromosome long arm gene complement is associated with sperm DNA damage.. Genome Biol.

[37] Lewis SE (2007). Is sperm evaluation useful in predicting human fertility?. Reproduction.

[38] Lefievre L, Bedu-Addo K, Conner SJ, Machado-Oliveira GS, Chen Y (2007). Counting sperm does not add up any more: time for a new equation?. Reproduction.

[39] de Mateo S, Ramos L, van der Vlag, de Boer P, Oliva R (2011). Improvement in chromatin maturity of human spermatozoa selected through density gradient centrifugation.. Int J Androl.

[40] Sousa AP, Amaral A, Baptista M, Tavares R, Caballero CP (2011). Not all sperm are equal: functional mitochondria characterize a subpopulation of human sperm with better fertilization potential.. PLoS One.

[41] Chandley AC, Goetz P, Hargreave TB, Joseph AM, Speed RM (1984). On the nature and extent of XY pairing at meiotic prophase in man.. Cytogenet Cell Genet.

[42] Heller CH, Clermont Y (1964). Kinetics of the germinal epithelium in man.. Recent Prog Horm Res.

[43] van der Laan R, Uringa EJ, Wassenaar E, Hoogerbrugge JW, Sleddens E (2004). Ubiquitin ligase Rad18Sc localizes to the XY body and to other chromosomal regions that are unpaired and transcriptionally silenced during male meiotic prophase.. J Cell Sci.

[44] Richler C, Ast G, Goitein R, Wahrman J, Sperling R (1994). Splicing components are excluded from the transcriptionally inactive XY body in male meiotic nuclei.. Mol Biol Cell.

[45] Ashley T, Gaeth AP, Creemers LB, Hack AM, de Rooij DG (2004). Correlation of meiotic events in testis sections and microspreads of mouse spermatocytes relative to the mid-pachytene checkpoint.. Chromosoma.

[46] Oliver-Bonet M, Ko E, Martin RH (2005). Male infertility in reciprocal translocation carriers: the sex body affair.. Cytogenet Genome Res.

[47] Burgoyne PS, Mahadevaiah SK, Turner JM (2009). The consequences of asynapsis for mammalian meiosis.. Nat Rev Genet.

[48] Solari AJ, Rey Valzacchi G (1997). The prevalence of a YY synaptonemal complex over XY synapsis in an XYY man with exclusive XYY spermatocytes.. Chromosome Res.

[49] Speed RM, Faed MJ, Batstone PJ, Baxby K, Barnetson W (1991). Persistence of two Y chromosomes through meiotic prophase and metaphase I in an XYY man.. Hum Genet.

[50] Hall H, Hunt P, Hassold T (2006). Meiosis and sex chromosome aneuploidy: how meiotic errors cause aneuploidy; how aneuploidy causes meiotic errors.. Curr Opin Genet Dev.

[51] Blanco J, Egozcue J, Vidal F (2001). Meiotic behaviour of the sex chromosomes in three patients with sex chromosome anomalies (47,XXY, mosaic 46,XY/47,XXY and 47,XYY) assessed by fluorescence in-situ hybridization.. Hum Reprod.

[52] Navarro-Costa P, Plancha CE, Goncalves J (2010). Genetic dissection of the AZF regions of the human Y chromosome: thriller or filler for male (in)fertility?. J Biomed Biotechnol.

[53] Oates RD, Silber S, Brown LG, Page DC (2002). Clinical characterization of 42 oligospermic or azoospermic men with microdeletion of the AZFc region of the Y chromosome, and of 18 children conceived via ICSI.. Hum Reprod.

[54] Navarro-Costa P, Goncalves J, Plancha CE (2010). The AZFc region of the Y chromosome: at the crossroads between genetic diversity and male infertility.. Hum Reprod Update.

[55] Skaletsky H, Kuroda-Kawaguchi T, Minx PJ, Cordum HS, Hillier L (2003). The male-specific region of the human Y chromosome is a mosaic of discrete sequence classes.. Nature.

[56] Hughes JF, Skaletsky H, Pyntikova T, Graves TA, van Daalen SK (2010). Chimpanzee and human Y chromosomes are remarkably divergent in structure and gene content.. Nature.

[57] Hammer MF, Woerner AE, Mendez FL, Watkins JC, Cox MP (2010). The ratio of human X chromosome to autosome diversity is positively correlated with genetic distance from genes.. Nat Genet.

[58] Bustamante CD, Ramachandran S (2009). Evaluating signatures of sex-specific processes in the human genome.. Nat Genet.

[59] Dupanloup I, Pereira L, Bertorelle G, Calafell F, Prata MJ (2003). A recent shift from polygyny to monogamy in humans is suggested by the analysis of worldwide Y-chromosome diversity.. J Mol Evol.

[60] Dixson AF, Anderson MJ (2004). Sexual behavior, reproductive physiology and sperm competition in male mammals.. Physiol Behav.

[61] Koopman P, Ashworth A, Lovell-Badge R (1991). The ZFY gene family in humans and mice.. Trends Genet.

[62] Song R, Ro S, Michaels JD, Park C, McCarrey JR (2009). Many X-linked microRNAs escape meiotic sex chromosome inactivation.. Nat Genet.

[63] Macklon NS, Geraedts JP, Fauser BC (2002). Conception to ongoing pregnancy: the ‘black box’ of early pregnancy loss.. Hum Reprod Update.

[64] Silber SJ (2000). Microsurgical TESE and the distribution of spermatogenesis in non-obstructive azoospermia.. Hum Reprod.

[65] Rowley MJ, Heller CG (1971). Quantitation of the cells of the seminiferous epithelium of the human testis employing the sertoli cell as a constant.. Z Zellforsch Mikrosk Anat.

[66] Zhengwei Y, Wreford NG, Royce P, de Kretser DM, McLachlan RI (1998). Stereological evaluation of human spermatogenesis after suppression by testosterone treatment: heterogeneous pattern of spermatogenic impairment.. J Clin Endocrinol Metab.

[67] McVicar CM, O'Neill DA, McClure N, Clements B, McCullough S (2005). Effects of vasectomy on spermatogenesis and fertility outcome after testicular sperm extraction combined with ICSI.. Hum Reprod.

[68] Hassold T, Judis L, Chan ER, Schwartz S, Seftel A (2004). Cytological studies of meiotic recombination in human males.. Cytogenet Genome Res.

[69] Oliver-Bonet M, Turek PJ, Sun F, Ko E, Martin RH (2005). Temporal progression of recombination in human males.. Mol Hum Reprod.

[70] Barlow AL, Benson FE, West SC, Hulten MA (1997). Distribution of the Rad51 recombinase in human and mouse spermatocytes.. EMBO J.

[71] Johnsen SG (1970). Testicular biopsy score count–a method for registration of spermatogenesis in human testes: normal values and results in 335 hypogonadal males.. Hormones.

[72] Peters AH, Plug AW, van Vugt MJ, de Boer P (1997). A drying-down technique for the spreading of mammalian meiocytes from the male and female germline.. Chromosome Res.

[73] Heyting C, Dietrich AJ (1991). Meiotic chromosome preparation and protein labeling.. Methods Cell Biol.

[74] Jao CY, Salic A (2008). Exploring RNA transcription and turnover in vivo by using click chemistry.. Proc Natl Acad Sci U S A.

[75] Evans EP, Breckon G, Ford CE (1964). An air-drying method for meiotic preparations from mammalian testes.. Cytogenetics.

[76] van der Heijden GW, Dieker JW, Derijck AA, Muller S, Berden JH (2005). Asymmetry in histone H3 variants and lysine methylation between paternal and maternal chromatin of the early mouse zygote.. Mech Dev.

