# Supplementary figures and images for: Human Male Meiotic Sex Chromosome Inactivation
Source: PLoS One. 2012 Feb 15;7(2):e31485. doi: 10.1371/journal.pone.0031485 (PMC3280304; doi:10.1371/journal.pone.0031485)

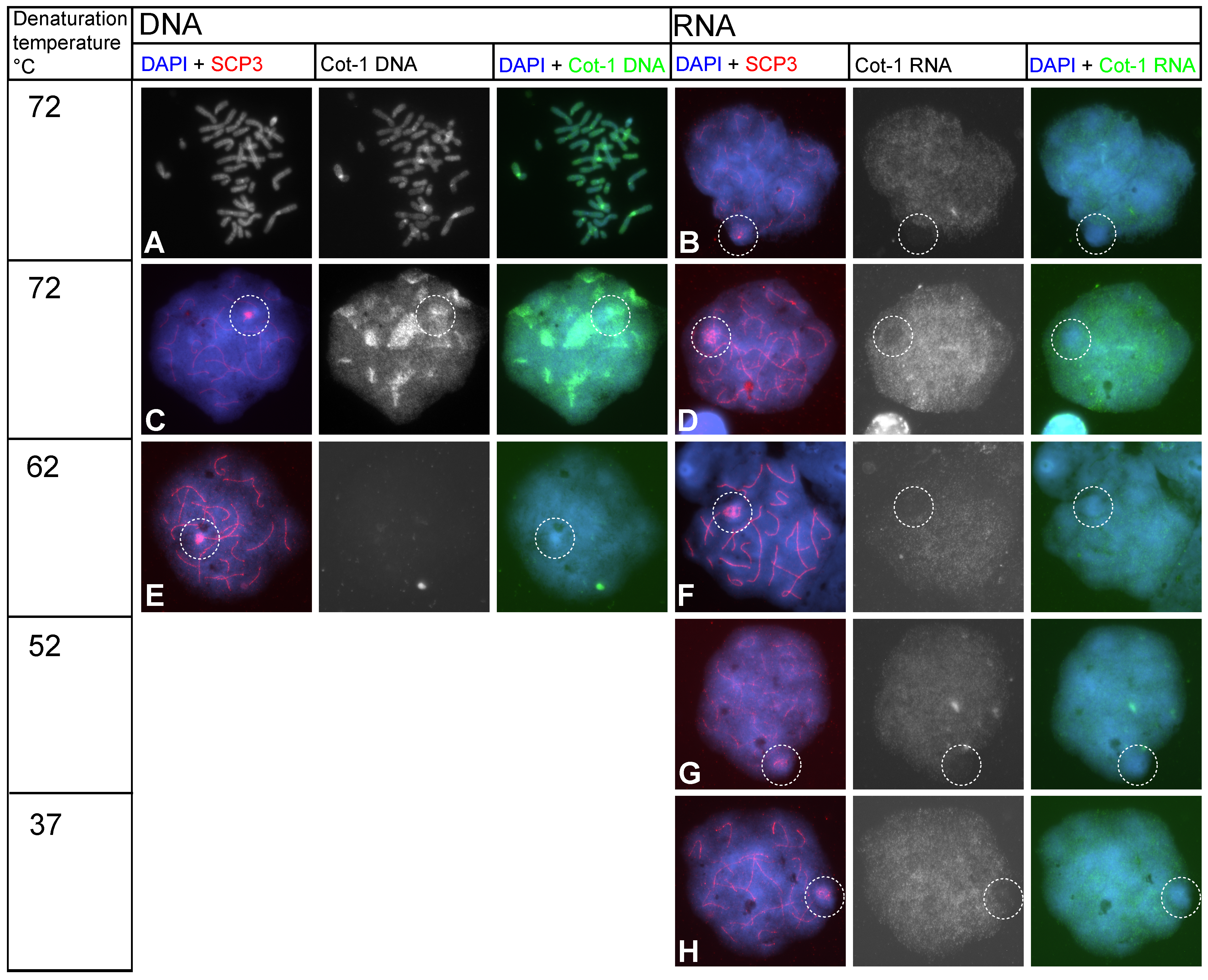

Supplement: Figure S2 — Validation of RNA FISH signals at different denaturation temperatures. B–H The dotted circle indicates the XY body. A Lymphocyte metaphase. After DNA FISH the Cot-1 probe hybridizes to all chromosomes and showed more intense staining at the centromeric heterochromatin. B Pachytene spermatocyte nucleus. After DNA FISH the Cot-1 probe hybridized to all autosomal chromosomes and showed decreased hybridization signal for X,Y chromatin. C RNAse-treated pachytene spermatocyte nucleus. After DNA FISH the Cot-1 probe hybridized to all chromosomes with more intense staining at the centromeric heterochromatin. D Pachytene spermatocyte nucleus. After RNA FISH at 72°C the Cot-1 probe hybridized to all autosomal chromosomes and showed decreased hybridization signals for XY chromatin. E RNAse treated pachytene spermatocyte nucleus. After DNA FISH at 62°C the Cot-1 probe was not able to hybridize. F–H Pachytene spermatocyte nucleus. After RNA FISH at 62°C, 52°C and 37°C respectively, the Cot-1 probe hybridized to all autosomal chromosomes and showed decreased hybridization signals for the XY chromatin. (TIF) [file pone.0031485.s002.tif]

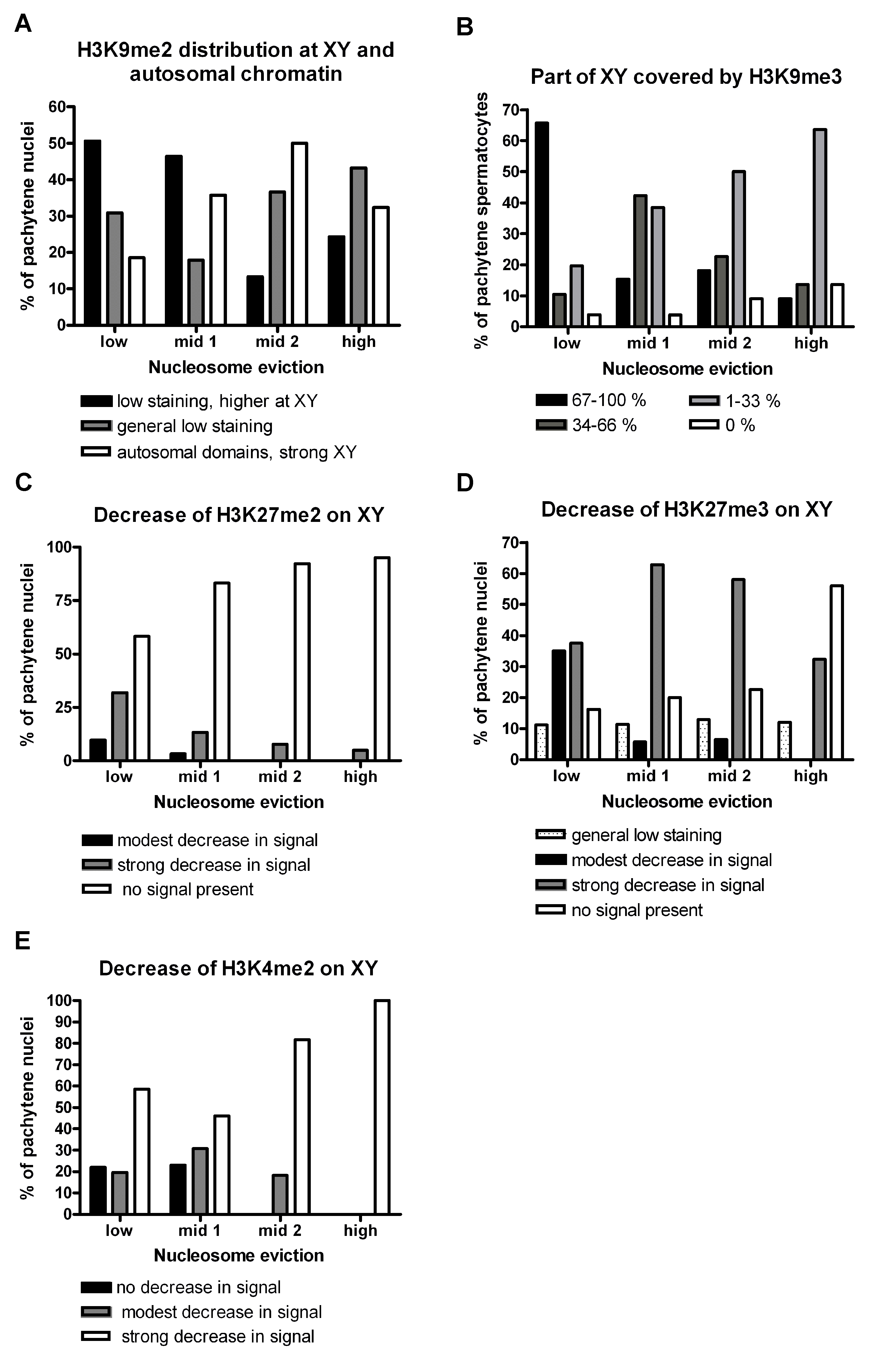

Supplement: Figure S3 — Graphical representation of histone dynamics at the XY chromatin in late pachytene spermatocytes. A–E Late pachytene nuclei were assigned to four groups, ranging from a low to a high degree of nucleosome eviction (see Figure 1B) (for probands included and number of nuclei see Table 2). Histone characteristics and histone N-tail modifications were subjectively determined for the XY body and in A for autosomal chromatin as well: A Nuclei were categorized into three goups (bars). ‘Low staining, higher at XY’ indicates at the observation of low, even autosomal staining with more intense staining at the XY body. ‘General low staining’ indicates at an overall low staining including the XY body. ‘Autosomal domains, strong XY’ points at the observation of a heterochromatin staining pattern on autosomal chromosomes and overall staining of the XY body. B The part of the XY chromatin stained was determined and arranged into four groups (bars). C–E The decrease in signal for the XY chromatin, compared to the autosomal chromatin, was determined and categorized into classes (bars). Statistical analysis by Chi-square; A: χ2 = 21.98 df 6, p<0.01, B: χ2 = 47.87 df 9, p<0.001 (Abcam antibody), C: χ2 = 29.2 df 6, p<0.001, D: χ2 = 40.2 df 9, p<0.001, E: χ2 = 10.9 df 6, p = 0.21 (Abcam antibody). (TIF) [file pone.0031485.s003.tif]
