# Supplementary material for: Human Male Meiotic Sex Chromosome Inactivation
Source: PLoS One. 2012 Feb 15;7(2):e31485. doi: 10.1371/journal.pone.0031485 (PMC3280304; doi:10.1371/journal.pone.0031485)
Supplement: Table S1 — Proband details. (DOC) [file pone.0031485.s004.doc]

**Table S1. Proband details.**

| **Patient/control** | **Age** | **Diagnosis** | **Duration vasectomy (years)** | **FSH level (IU/ml)** | **ICSI cycles** | **Pregnancy (at ICSI cycle no.)** | **Pach/ sperm** | **Sperm per 100 SCs** |
| --- | --- | --- | --- | --- | --- | --- | --- | --- |
| P1 | 33 | OA | n/a | 7 | 2 | Miscarriage (#1), Yes (#2) | 0.97 | 113 |
| P2 | 32 | NOA | n/a | 20.6 | 5 | No (#1-5) | 0.48 | 16 |
| P3 | 38 | NOA | n/a | 20.4 | 1 | No (#1) | n.d. | n.d. |
| P4 | 28 | NOA | n/a | 23 | 1 | No (#1) | 0.60 | 84 |
| C1 | 43 | OA | 14 | ND | - | - | 0.33 | 71 |
| C2 | 48 | OA | 8 | 5.5 | 1 | Yes (#1) | 0.36 | 121 |
| C3 | 39 | OA | 5 | 6.4 | 3 | No (#1,2) Miscarriage (#3) | n.d. | n.d. |

Clinical data from patients (P) and controls (C). In every ICSI cycle several oocytes were fertilized and one or two best quality embryos were transferred to the uterus. Ongoing pregnancy is determined at 12 weeks after embryo transfer. Treatment had not yet started for control 1. Pachytene/sperm ratio (pach/sperm) and the number of sperm per 100 Sertoli cells (SCs) were determined in a Giemsa stained sample of the homogenized fresh biopsy.
