# Supplementary material for: Human Male Meiotic Sex Chromosome Inactivation
Source: PLoS One. 2012 Feb 15;7(2):e31485. doi: 10.1371/journal.pone.0031485 (PMC3280304; doi:10.1371/journal.pone.0031485)
Supplement: Table S2 — Literature data on meiotic expression of several MSY/AZFa,b,c genes. (DOC) [file pone.0031485.s005.doc]

**Table S2. Literature data on meiotic expression of several MSY/AZFa,b,c genes**

| Gene | AZFa,b,c | Expression | | | | | | Reference |
| --- | --- | --- | --- | --- | --- | --- | --- | --- |
|  |  | Spermatogonia | | Spermatocytes | | Spermatids | |  |
|  |  | RNA | Protein | RNA | Protein | RNA | Protein |  |
| DDX3Y (DBY) | a | +,+ | + | + | +/- | +,+ | - | [1,2]: present in some leptotenes and zygotenes absent in pachytene |
| USP9Y | a |  |  |  |  |  | + | [3]:not essential for fertility |
| RBMY | b |  | + |  | + |  | + | [4-6] |
| SMCY (JARID1D) | b | + |  | + |  | + |  | [1] |
| DAZ | c | +,+ | +,+ | +/-, +/- | +/- | - | - | [7,8]: high in leptotene, zygotene, low in late pachytene [9,10] |
| CDY1/2 | b, c | - | - | - | - | + | + | [11,12] |
| SRY | Y | + |  | +/- |  | + |  | [13]: low in early first prophase, absent in pachytene |
| RPS4Y1/2 | Y,b | + |  | + |  | + |  | [1,11] |
| TSPY | Y |  | +,+ |  | +/-,+/- |  | +/- | [14,15]: gets lower in early pachytene |

DAZ, SRY and TSPY follow the MSCI pattern. The alleviation of MSCI is most convincing for RBMY. No variability between nuclei is indicated. No data for ZFY have been found.

**References**

(1) Yao C, Wang Z, Zhou Y, Xu W, Li Q, et al. (2010) A study of Y chromosome gene mRNA in human ejaculated spermatozoa. Mol Reprod Dev 77: 158-166.

(2) Jaroszynski L, Zimmer J, Fietz D, Bergmann M, Kliesch S, et al. (2010) Translational control of the AZFa gene DDX3Y by 5'UTR exon-T extension. Int J Androl .

(3) Vogt PH, Falcao CL, Hanstein R, Zimmer J. (2008) The AZF proteins. Int J Androl 31: 383-394.

(4) Elliott DJ, Millar MR, Oghene K, Ross A, Kiesewetter F, et al. (1997) Expression of RBM in the nuclei of human germ cells is dependent on a critical region of the Y chromosome long arm. Proc Natl Acad Sci U S A 94: 3848-3853.

(5) Elliott DJ, Oghene K, Makarov G, Makarova O, Hargreave TB, et al. (1998) Dynamic changes in the subnuclear organisation of pre-mRNA splicing proteins and RBM during human germ cell development. J Cell Sci 111 ( Pt 9): 1255-1265.

(6) Venables JP, Elliott DJ, Makarova OV, Makarov EM, Cooke HJ, et al. (2000) RBMY, a probable human spermatogenesis factor, and other hnRNP G proteins interact with Tra2beta and affect splicing. Hum Mol Genet 9: 685-694.

(7) Menke DB, Mutter GL, Page DC. (1997) Expression of DAZ, an azoospermia factor candidate, in human spermatogonia. Am J Hum Genet 60: 237-241.

(8) Reijo RA, Dorfman DM, Slee R, Renshaw AA, Loughlin KR, et al. (2000) DAZ family proteins exist throughout male germ cell development and transit from nucleus to cytoplasm at meiosis in humans and mice. Biol Reprod 63: 1490-1496.

(9) Szczerba A, Jankowska A, Andrusiewicz M, Karczewski M, Turkiewicz W, et al. (2004) Distribution of the DAZ gene transcripts in human testis. Folia Histochem Cytobiol 42: 119-121.

(10) Huang WJ, Lin YW, Hsiao KN, Eilber KS, Salido EC, et al. (2008) Restricted expression of the human DAZ protein in premeiotic germ cells. Hum Reprod 23: 1280-1289.

(11) Navarro-Costa P, Plancha CE, Goncalves J. (2010) Genetic dissection of the AZF regions of the human Y chromosome: thriller or filler for male (in)fertility? J Biomed Biotechnol 2010: 936569.

(12) Lahn BT, Tang ZL, Zhou J, Barndt RJ, Parvinen M, et al. (2002) Previously uncharacterized histone acetyltransferases implicated in mammalian spermatogenesis. Proc Natl Acad Sci U S A 99: 8707-8712.

(13) Modi D, Shah C, Sachdeva G, Gadkar S, Bhartiya D, et al. (2005) Ontogeny and cellular localization of SRY transcripts in the human testes and its detection in spermatozoa. Reproduction 130: 603-613.

(14) Schnieders F, Dork T, Arnemann J, Vogel T, Werner M, et al. (1996) Testis-specific protein, Y-encoded (TSPY) expression in testicular tissues. Hum Mol Genet 5: 1801-1807.

(15) Lau YF, Li Y, Kido T. (2011) Role of the Y-located putative gonadoblastoma gene in human spermatogenesis. Syst Biol Reprod Med 57: 27-34.
