# Supplementary material for: Human Male Meiotic Sex Chromosome Inactivation
Source: PLoS One. 2012 Feb 15;7(2):e31485. doi: 10.1371/journal.pone.0031485 (PMC3280304; doi:10.1371/journal.pone.0031485)
Supplement: Methods S1 — Supporting methods. (DOC) [file pone.0031485.s006.doc]

**Methods S1**

**Optimization of Cot-1 RNA FISH in combination with immunofluorescence**

To verify genome coverage of the preparation of Cot-1 RNA, DNA FISH was performed on spread human peripheral blood lymphocyte chromosomes. All chromosomes were covered with a Cot-1 signal, with an emphasis on constitutive centric heterochromatin (Figure S2A). DNA- and RNA FISH differ in initial denaturation temperature and in stringency washes (Material and Methods). When performing DNA FISH for Cot-1 on pachytene spermatocytes, an RNA FISH pattern emerged at an increased intensity of labelling compared with RNA FISH with this probe at the typical hybridisation temperature of 37°C (compare Figure S2B, H). To investigate the relation between DNA and RNA hybridisation protocols further, we designed the following experiment: We performed DNA/RNA FISH at a gradient of denaturation temperatures (72, 62, 52, 42 and 37°C). DNA is assumed not to denature at 62°C and below. At every temperature one slide was treated with RNase and one slide was not. Slides that were treated with RNAse were post-hybridization subjected to stringency washes for DNA FISH (see below). Slides not treated with RNase were subjected to RNA FISH stringency washes (Materials and Methods). At “denaturation temperatures” below 72°C, the Cot-1 probe was denatured in advance at 80°C for 10 minutes to be sure the probe would be single-stranded.

At 72°C without RNase treatment, we observed even staining throughout the nucleus with a decreased or absent signal on XY (Figure S2D). After RNase treatment, we observed complete coverage of all chromosomes with more intense areas around the centromeres (Figure S2C). Also the X and Y chromatin was stained with Cot-1 and both the X and Y chromosomes contained a Cot-1 intense region which was clearly visible in early pachytene nuclei (not shown). At 62°C and 52°C without RNAse treatment, we observed again the supposed RNA signal albeit at a lower intensity (Figure S2F, G). After RNase treatment, we could not observe any signal at 62°C (Figure S2E) or lower temperatures (not shown). In this condition, the RNA was removed and the DNA not denatured, hence no hybridisation followed. This result proved to us that nuclei denatured at 62°C and untreated with RNase, did show an RNA signal. When comparing signal intensities between RNA FISH at 72°C and 37°C the signal was more intense at 72°C when slides were subjected to RNA-specific stringency washes (compare Figure S2B,D and H). We therefore decided to perform succeeding RNA FISH experiments at 72°C (Materials and Methods, Results and Figure 5)

**Cot-1 DNA FISH**

RNAse A (Roche diagnostics) treatment was performed for two hours at 37°C (100 ug/ml in PBS). Cot-1 DNA FISH was performed with biotin-labelled Cot-1 DNA probes as described in Materials and Methods. 300 ng DNA was placed on a slide, denatured at 72°C and hybridized overnight at 37°C in a humid box. The next day, slides were subjected to several stringency washes specific for DNA FISH starting with two times (1 minute) 0.4x SSC/0.1% Tween 20 at 68°C and subsequently once in 2x SSC followed by a wash in 4x SSC/0.1% Tween 20. Then slides were blocked for 10 minutes in a humid box at 37°C. Probe detection was carried out using avidine-FITC followed by goat anti-avidine FITC. Both antibodies were incubated for 30 minutes at 37°C. In between and after the incubations, slides were washed three times in 4x SSC/0.1% Tween 20. Before IF, slides were once washed in PBS at room temperature. The first antibody was diluted in PBS and incubated for 1 hour at 37°C followed by three PBS washes. The secondary antibody was incubated for 30 minutes at 37°C followed by 3 PBS washes. DAPI staining was performed and slides were mounted with Vectashield.
